# MMPs-related risk model identification and SAA1 promotes clear cell renal cell carcinoma migration via ERK-AP1-MMPs axis

**DOI:** 10.1038/s41598-024-59112-5

**Published:** 2024-04-24

**Authors:** Haotian Wei, Yajun Li, Jian Zhang, Chenglong Xu, Dadong Wei, Changyi Quan, Shimiao Zhu

**Affiliations:** 1 Department of Urology, Tianjin Institute of Urology, The Second Hospital of Tianjin Medical University, Tianjin, China; 2 Department of Urology, Affiliated Hospital of Chifeng University, Chifeng, China; 3 Department of Orthopedics, First Affiliated Hospital of Dalian Medical University, Dalian, China

**Keywords:** Cancer, Computational biology and bioinformatics, Molecular biology, Urology

## Abstract

Matrix Metalloproteinases (MMPs) have been demonstrated to be essential in facilitating the migration and metastasis of clear cell renal cell carcinoma (ccRCC). However, the ability of the MMP family to predict clinical outcomes and guide optimal therapeutic strategies for ccRCC patients remains incompletely understood. In this investigation, we initially conducted a thorough examination of the MMP family in pan-cancer. Notably, MMPs exhibited distinctive significance in ccRCC. Following this, we undertook an extensive analysis to evaluate the clinical value of MMPs and potential mechanisms by which MMPs contribute to the progression of ccRCC. A novel stratification method and prognostic model were developed based on MMPs in order to enhance the accuracy of prognosis prediction for ccRCC patients and facilitate personalized treatment. By conducting multi-omics analysis and transcriptional regulation analysis, it was hypothesized that SAA1 plays a crucial role in promoting ccRCC migration through MMPs. Subsequently, in vitro experiments confirmed that SAA1 regulates ccRCC cell migration via the ERK-AP1-MMPs axis. In conclusion, our study has explored the potential value of the MMP family as prognostic markers for ccRCC and as guides for medication regimens. Additionally, we have identified SAA1 as a crucial factor in the migration of ccRCC.

## Introduction

Renal cell carcinoma (RCC), one of the most common genitourinary tumors, has long been recognized as a major threat. According to the official statistical data, the incidence of RCC represents about 3–5% of all malignancies^1^. With the continuous advances in medical imaging technology, incidental RCC has accounted for 60–70% of all RCC, although 25–30% of patients still have metastases at initial diagnosis. About one-fifth of patients who undergo radical nephrectomy will show recurrence or distant metastasis^2^. Given the absence of efficient therapies, patients diagnosed with advanced clear cell RCC (ccRCC) face a bleak outlook, with a mere 10% chance of surviving for five years^3^. Therefore, to address these factors and guide personalized treatment, it is imperative to identify a stable molecular model to accurately predict prognosis and select sensitive drugs.

Considering the highly metastatic characteristic of RCC and the refractory characteristic of advanced RCC, we focused on the enzymes which assist the extracellular matrix (ECM) formation and degradation, such as matrix metalloproteinases (MMPs)^4^. MMPs are members of zinc-dependent endopeptidases family that remodel the ECM and have been demonstrated to have implications on tumor migration, invasion, metastasis, and the establishment of the tumor microenvironment^5–10^. MMPs dysregulation is common in tumors, and more and more studies are focusing on the prognostic value of MMPs^11–17^. In the domain of ccRCC-related research, investigations pertaining to the MMP family have predominantly focused on MMP2, MMP7, and MMP9^18–21^. For instance, prior research have demonstrated the utility of MMP-7 as a marker, with its levels being independently associated with reduced disease-specific survival in ccRCC^22^. MMP9 has also been recognized as a negative prognostic factor for RCC and an influential factor for tumor metastasis in ccRCC^23^. However, the examination of other members of the MMP family in ccRCC remains limited. Furthermore, conflicting data exists regarding the involvement of various MMP family members in tumorigenesis^17,24^. Consequently, a comprehensive analysis encompassing the entire MMP family in ccRCC is imperative from a holistic perspective. Moreover, the potential of these members to prognosticate clinical outcomes and dictate optimal therapeutic strategies for patients with ccRCC remains enigmatic. As a result, the formulation of a risk stratification framework and prognostic model for ccRCC based on MMP family members assumes paramount significance. The aim of our study was to fill this research gap.

## Results

### Pan-cancer characteristics of MMPs

**Figure 1:**
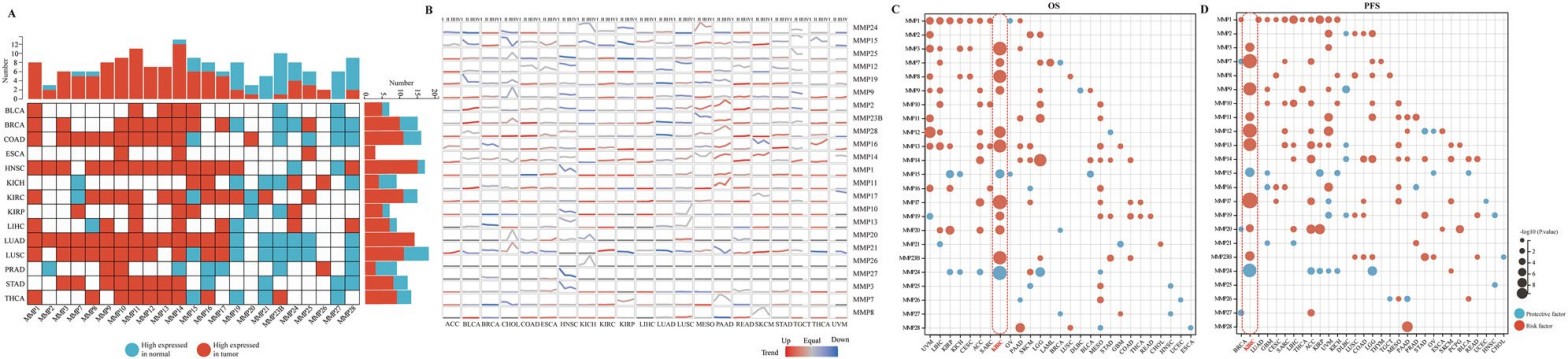
Pan-cancer analysis of MMPs. (**A**) Expression pattern among MMPs in human tumors. Blue represents high expressed in normal tissues, and red represents high expressed in tumor tissues. (**B**) Expression tendency of MMPs from early stage to advanced stage. (**C**,**D**) Survival landscape among MMPs in human tumors. Blue represents protective factor, and red represents risk factor.

In order to enhance understanding of the expression patterns and prognostic significance of MMPs, a comprehensive analysis was undertaken in pan-cancer. Initially, we conducted a comparison of the expression of 23 MMPs between tumor and normal samples. The findings demonstrated significant differential expression of the majority of MMPs between tumor and normal tissues (Fig. 1A and Supplementary Fig. 1A). Interestingly, MMP1-MMP17 were mostly highly expressed in tumor tissues, while MMP19-MMP28 were mostly highly expressed in normal tissues. Furthermore, our findings indicate that the expression levels of MMPs exhibit distinct patterns in relation to clinical stage in different types of cancer (Fig. 1B). Specifically, in KIRC, READ, and THCA, there is a tendency for the expression levels of most MMPs to increase with clinical staging, whereas in HNSC and LUAD, the expression levels of most MMPs tend to decrease with clinical staging.

Subsequently, we conducted univariate Cox regression analysis in order to identify genes that potentially possess prognostic implications. It is noteworthy to emphasize that the prognostic significance of MMPs varies across different cancer types (Fig. 1C,D). Significantly, a considerable proportion of MMPs (12/23 for overall survival. (OS), 11/23 for progression-free survival (PFS)) demonstrated prognostic significance for ccRCC. Specifically, MMP3, MMP7, MMP8, MMP9, MMP12, MMP13, MMP17, MMP19, MMP20, and MMP23B were identified as independent risk factors for ccRCC, while MMP15 and MMP24 were identified as independent protective factors for ccRCC (Supplementary Fig. 1B,C).

Finally, the presence of copy number variation (CNV) mutations in MMPs was documented (Supplementary Fig. 2A,B). Notably, a substantial prevalence of amplification CNV mutations was observed for MMP9, MMP16, and MMP24. Additionally, the frequencies of deletion CNV mutations were found to be significantly higher in TGCT, UCS, and OV. Moreover, a predominant inclination towards low-frequency single nucleotide variant (SNV) mutations was observed in pan-cancer (Supplementary Fig. 2C). However, it is worth mentioning that an elevated occurrence of SNV mutations was only observed in SKCM and UCEC.

### Consensus clustering analysis based on MMPs

**Figure 2:**
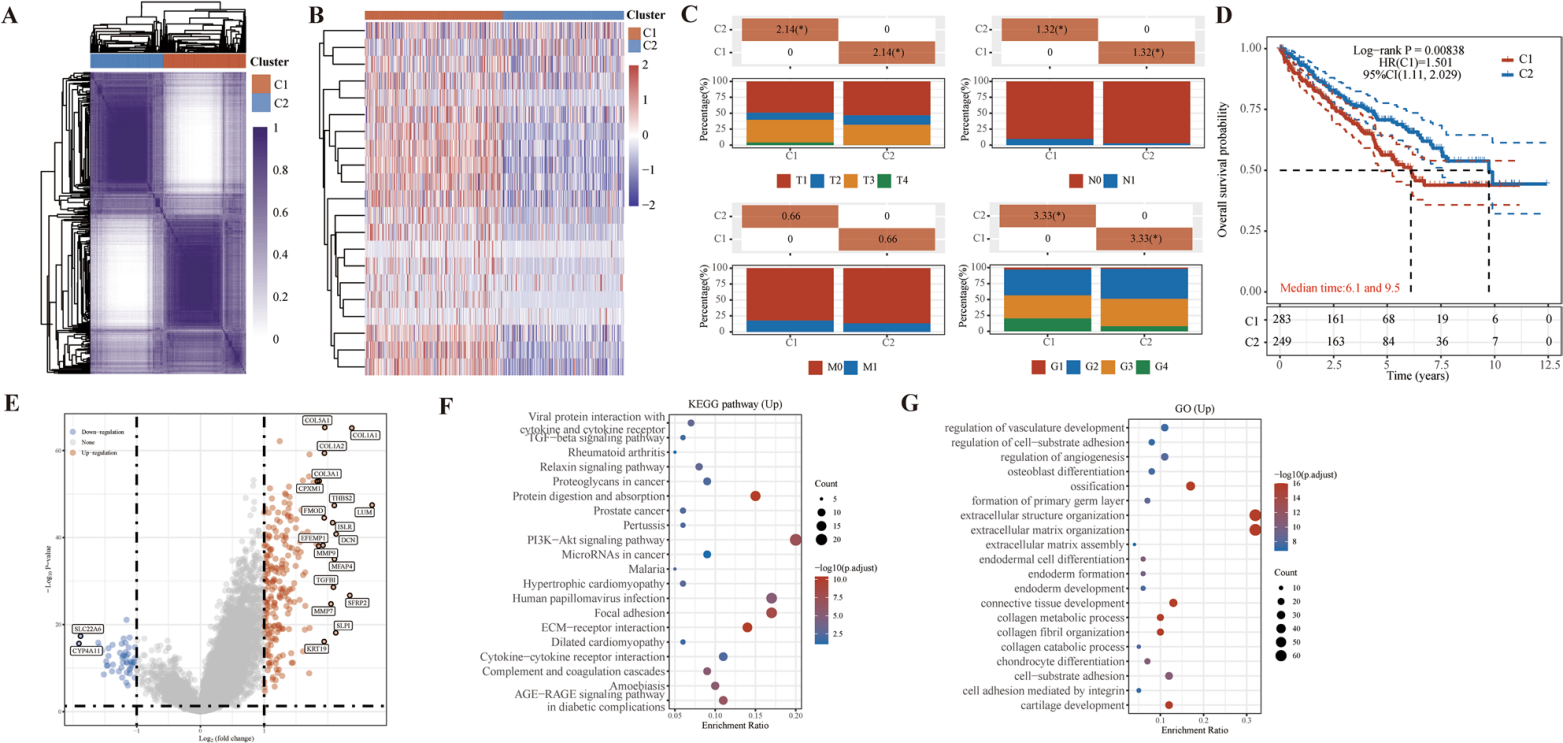
Identification of MMPs-related subtype. (**A**) Consensus clustering matrix of MMPs-related subtype. (**B**) Gene expression heat map of MMPs-related subtype. (**C**) The distribution of clinical characteristics (T stage, N stage, M stage, and Grade) of MMPs-related subtype. (**D**) KM curves between two clusters. (**E**) Volcano plot presenting the DEGs between two clusters. (**F**,**G**) Enrichment analysis of up-regulated DEGs.

In view of the potential role of MMPs in the development of ccRCC and its underlying mechanism, we pioneered molecular typing to ccRCC patients based on 23 MMPs and initially explored its clinical value. We classified ccRCC patients from the TCGA-KIRC cohort into cluster 1 (C1) and cluster 2 (C2) based on this gene set using a consensus clustering algorithm (Fig. 2A). And the two clusters were further identified by principal component analysis (Supplementary Fig. 3A), and gene expression heatmap (Fig. 2B). According to the heatmap, it was observed that cluster 1 exhibited a high expression of most MMPs, thereby characterizing it as a cluster with elevated MMPs activity. Noteworthy, cluster 2 demonstrated a greater prevalence of patients with low T stage, N stage, and grade (Fig. 2C and Supplementary Fig. 3B). Furthermore, this particular cluster displayed a significantly prolonged OS (Fig. 2D). The differentially expressed genes (DEGs) were then analyzed to explore the potential mechanisms that may contribute to poor prognosis. We found that DEGs were mainly highly expressed genes in cluster 1, including MMP7, MMP9, and TGFB1 (Fig. 2E). Enrichment analysis of DEGs highly expressed in cluster 1 revealed that cell adhesion-related pathways including Focal adhesion, ECM-receptor interaction, and extracellular matrix organization were significantly enriched (Fig. 2F,G and Supplementary Fig. 3C).

### Immune related analysis and drug sensitivity analysis

**Figure 3:**
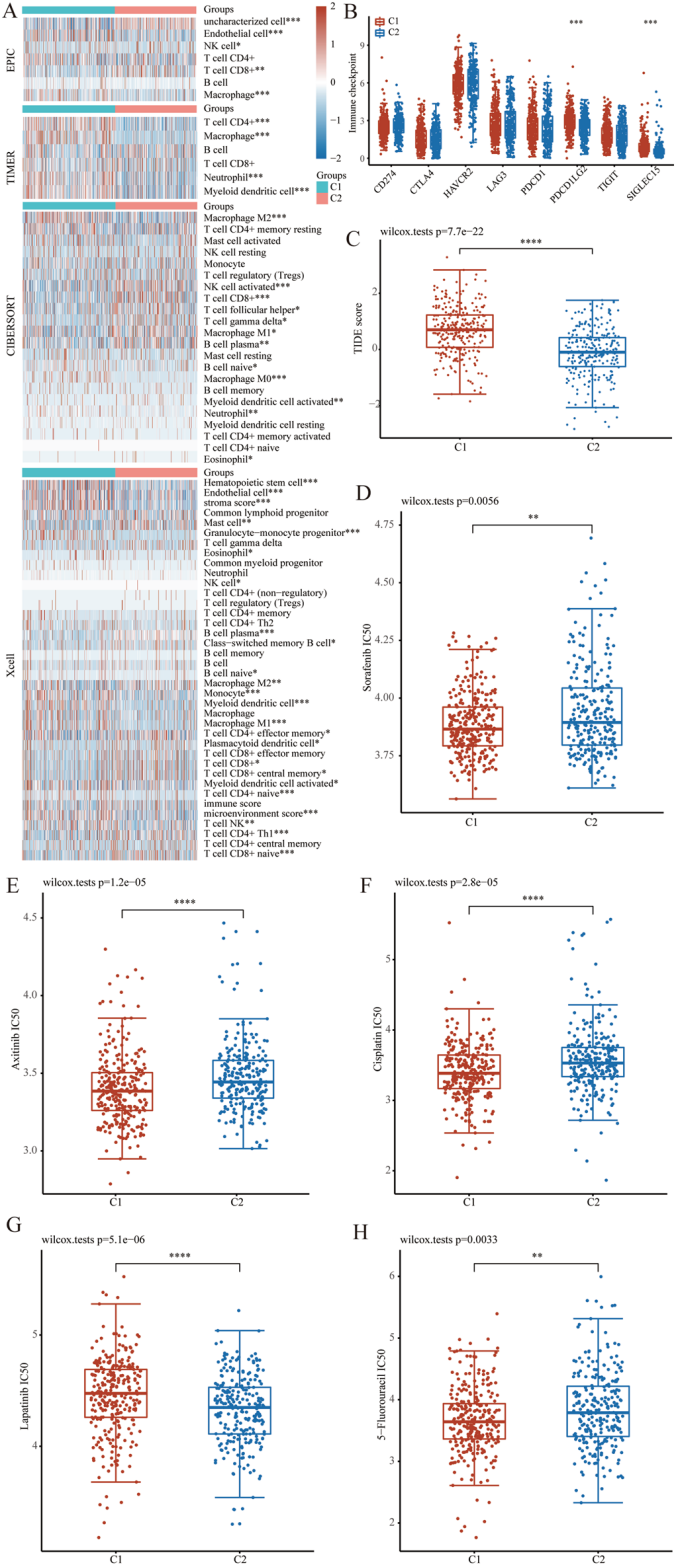
Immune-related analysis and drug sensitivity analysis of MMPs-related subtype. (**A**) Heatmap showed the difference in immune infiltration between two clusters. (**B**) The box plots encompassed the expression distribution of immune checkpoints genes between two clusters. (**C**) The distribution of immune response scores (TIDE scores) between two clusters. (**D**–**H**) The box plot illustrates the IC50 values of 5 commonly used drugs (sorafenib, axitinib, cisplation, 5-Fluorouracil, and lapatinib) between two clusters.

In order to enhance the assessment of the clinical value of this stratification method, additional analyses were performed, including immune-related analysis and drug sensitivity analysis. Initially, a thorough examination was undertaken utilizing various computational tools including TIMER, CIBERSORT, EPIC, and XCELL algorithm to identify variations in levels of immune cell infiltration (Fig. 3A). It is evident that substantial disparities exist in the tumor immune microenvironment between cluster 1 and cluster 2 patients. Of particular interest among these disparities is the discrepancy in levels of infiltration by M2 macrophages, which are found to be relatively higher in cluster 1. Subsequently, an examination was conducted on the expression of immune checkpoint genes within distinct subclusters, revealing that solely PDCD1LG2 and SIGLEC15 exhibited significantly elevated expression levels in cluster 1(Fig. 3B). Ultimately, the Tumor Immune Dysfunction and Exclusion (TIDE) algorithm was employed to forecast the responsiveness to immune checkpoint inhibitors (Fig. 3C). It was observed that cluster 1, characterized by high TIDE scores, may exhibit diminished effectiveness in response to immune checkpoint blockade therapy (ICB).

Given the significance of targeted therapy in the management of ccRCC and its promising potential, we conducted a comprehensive assessment of the responsiveness of distinct subclusters to common targeted and chemotherapeutic agents (Fig. 3D–H). Our findings revealed that cluster 1 exhibits greater sensitivity to most of the targeted drugs and chemotherapeutic agents including sorafenib, axitinib, cisplation and 5-Fluorouracil. Cluster 2 was only sensitive to lapatinib. Consequently, the stratification method developed utilizing MMPs not only enables prognostication of patients but also offers valuable guidance in selecting suitable medications.

### MMP-related prognostic risk model development and validation

**Figure 4:**
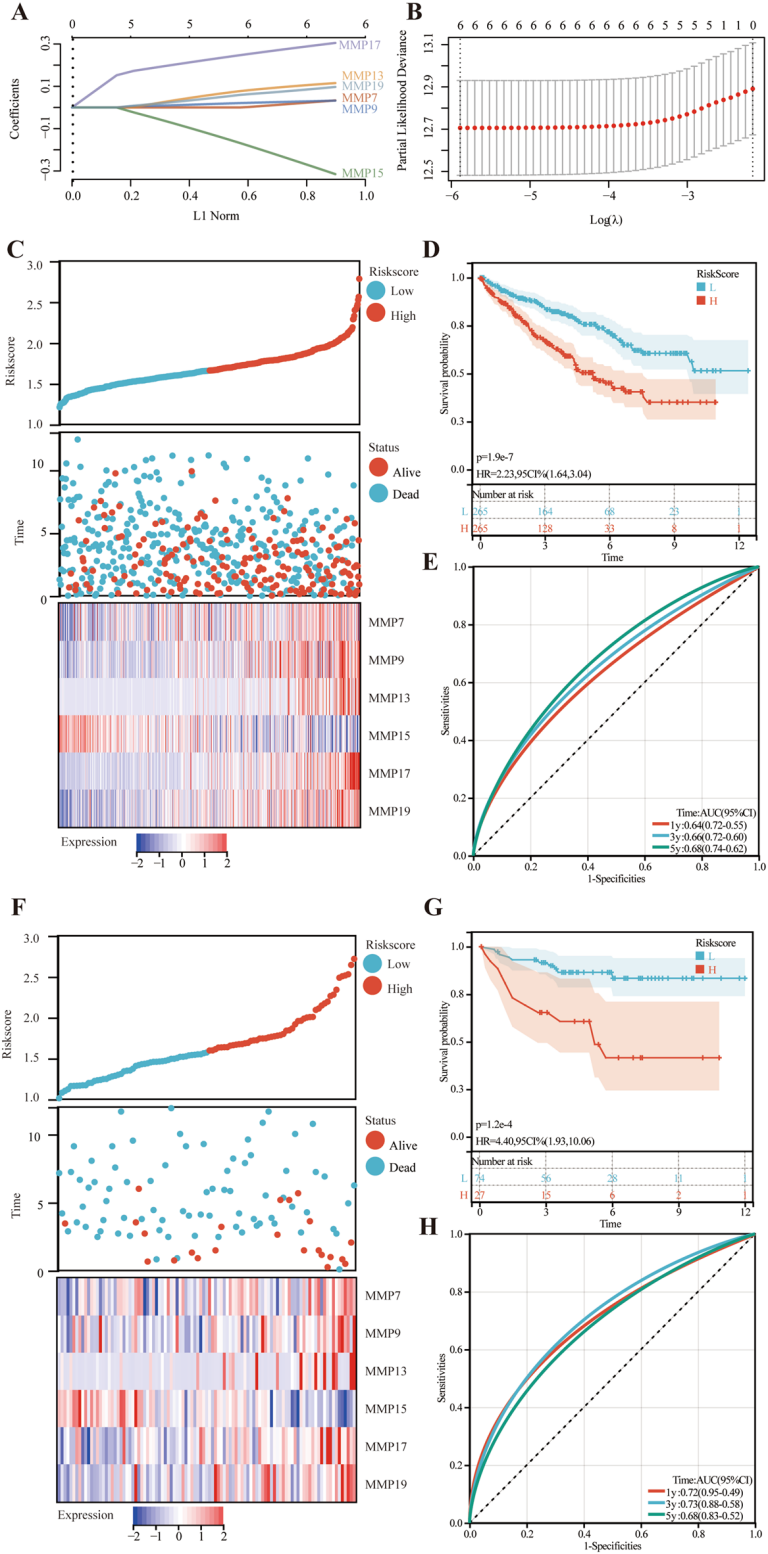
Establishment and validation of the MMP-related prognostic risk model. (**A**) LASSO coefficient profiles of the 6 MMPs. (**B**) The optimal tuning parameter in the LASSO model. (**C**) Scatterplot and heatmap presenting the risks core, survival time, survival status and 6 MMPs expression of the training set (TCGA-KIRC cohort). (**D**) KM curves between the high-risk group and low-risk group in the training set. (**E**) The 1-year, 3-year and 5-year ROC curves in the training set. (**F**) Scatterplot and heatmap presenting the risks core, survival time, survival status and 6 MMPs expression of the test set (E-MATB-1980 cohort). (**G**) KM curves between the high-risk group and low-risk group in the test set. (**H**) The 1-year, 3-year and 5-year ROC curves in the test set.

Based on the finding that MMPs were associated with ccRCC patient prognosis, we developed an MMP-related prognostic risk model to better predict patient prognosis. Combined with the results of the previous analysis, six differentially expressed MMPs with prognostic values were finally screened, including MMP7, MMP9, MMP13, MMP15, MMP17, and MMP19. We perform dimensionality reduction and construct prognostic models based on the LASSO regression algorithm in the TCGA-KIRC cohort. tenfold cross-validation results show that the model is optimal when the optimal adjustment parameter is 0.028 (Fig. 4A,B). The final generated prognostic model equation is as follows: Risk score = (0.0334) *MMP7 + (0.0323) *MMP9 + (0.1152) *MMP13 + (− 0.3149) *MMP15 + (0.3048) *MMP17 + (0.0969) *MMP19. Following the determination of the optimal risk score threshold, all patients were subsequently categorized into either the high-risk or low-risk group (Fig. 4C). The duration of OS was considerably shorter in the high-risk group compared to the low-risk group (Fig. 4D). The area under curves (AUCs) for the 1-, 3-, and 5-year periods were recorded as 0.64, 0.66, and 0.68, respectively (Fig. 4E). Furthermore, the risk model was validated using the independent E-MTAB-1980 cohort as a test set to confirm its predictive significance. The survival analysis exhibited a comparable pattern to previous findings, indicating a significantly reduced OS in the high-risk group (Fig. 4F,G). The AUCs for the 1-, 3-, and 5-year periods were recorded as 0.72, 0.73, and 0.68 in the validation cohort (Fig. 4H).

Then, we showed the association between risk score and clinicopathological characteristics using the Sankey diagram (Supplementary Fig. 4A). To assess the prognostic predictive value, a stratified subgroup analysis was performed according to more precisely to the clinicopathological features, and the results suggested that the MMP-related prognostic risk model has a reliable predictive ability (Supplementary Fig. 4B–E).

### The potential MMPs-driven mechanism to promote the progression of ccRCC

**Figure 5:**
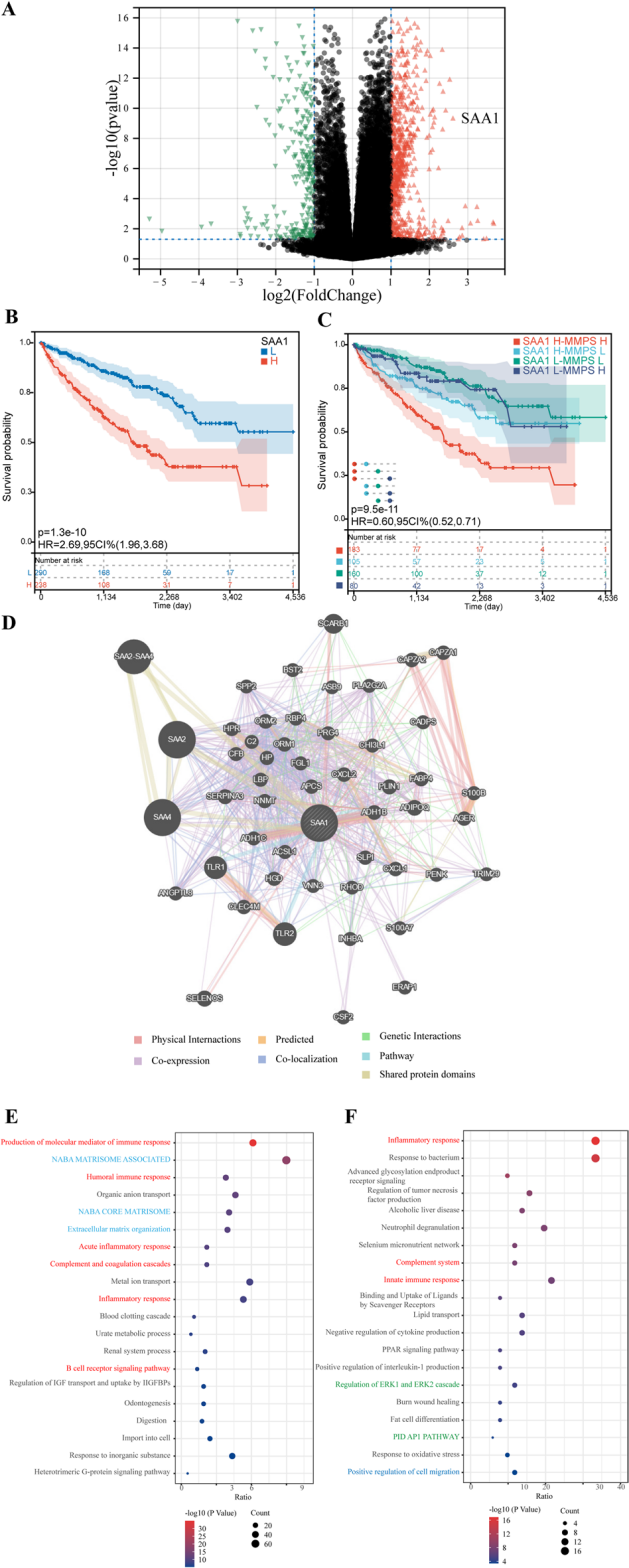
The potential mechanisms behind MMPs-driven ccRCC progression. (**A**) Volcano plot presenting the DEGs between the high-risk score group and low-risk score group. (**B**) KM curves between the high-SAA1 expression group and the low-SAA1 expression group. (**C**) KM curves among the SAA1-H MMPS-H group, SAA1-H MMPS-L group, SAA1-L MMPS-H group, and SAA1-L MMPS-L group. (**D**) The protein–protein interaction network of SAA1 based on the GeneMANIA database. (**E**) Enrichment analysis of upregulated DEGs in high-risk score group. (**F**) Enrichment analysis of SAA1-related genes.

Next, we examined the DEGs between the high-risk and low-risk groups in order to investigate the potential mechanisms that could be responsible for unfavorable prognosis. With a cut-off fold change of 2 and a *P*-value of 0.05, 619 genes are up-regulated and 214 genes are down-regulated (Fig. 5A). SAA1 was found to be one of the most significantly upregulated genes in the high-risk group. The presence of high expression of SAA1 also predicts a poor prognosis (Fig. 5B). To further explore the regulatory mechanisms of MMPs and SSA1, we further divided the high-risk score group and low-risk score group into four groups based on the expression of SAA1: the high-SAA1 expression and high-risk score (SAA1-H MMPS-H) group, the high-SAA1 expression and low-risk score (SAA1-H MMPS-L) group, the low-SAA1 expression and high-risk score (SAA1-L MMPS-H) group, and the low-SAA1 expression and low-risk score (SAA1-L MMPS-L) group. We repeated the survival analysis and found that the shortest OS was observed in the SAA1-H MMPS-H group (Fig. 5C). From this perspective, SAA1 may have a potential regulatory relationship with MMPs.

SAA1-related genes were selected by the GeneMANIA database (Fig. 5D). Enrichment analysis of DEGs revealed that cell adhesion/migration-related pathways and immune-related pathways were significantly enriched, such as NABA matrisome associated, NABA core matrisome, humoral immune response, and inflammatory response (Fig. 5E). Enrichment analysis of SAA1-related genes showed similar results that inflammatory response, innate immune response, and positive regulation of cell migration were significantly enriched (Fig. 5F). Furthermore, the enrichment analysis revealed significant associations between SAA1-related genes and the ERK and AP-1 signaling pathways, indicating that SAA1 may modulate the expression of MMPs through the regulation of the ERK/AP-1 axis.

**Figure 6:**
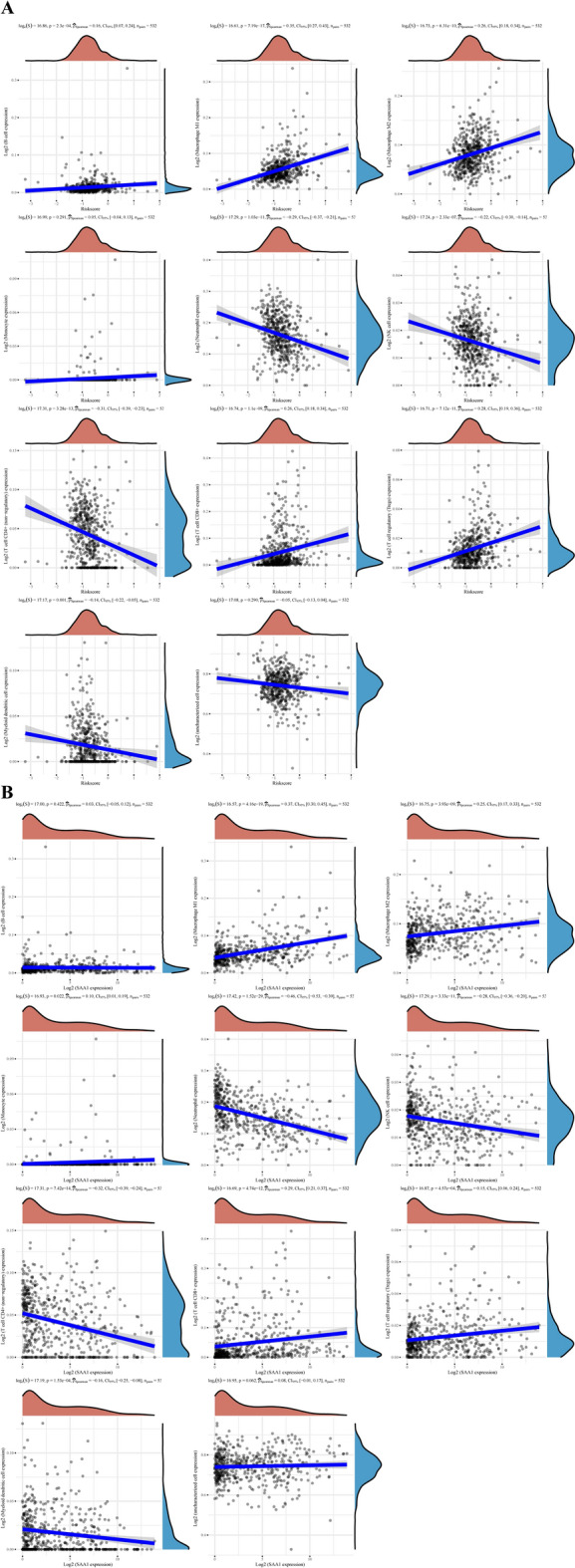
Analysis of immune infiltration. (**A**) The associations between risk scores and the levels of infiltration of different immune cells using the QUANTISEQ algorithms. (**B**) The associations between SAA1 expression and the levels of infiltration of different immune cells using the QUANTISEQ algorithms.

Additionally, we excavated the correlations between the risk score and immune cell infiltrations based on the QUANTOISEQ algorithms. The risk score positively correlated with the level of infiltration of B cell, M1 macrophage, M2 macrophage, CD8 + T cell, and T regulatory cell, and negatively correlated with the level of infiltration of neutrophil, NK cell, CD4 + T cell, and myeloid dendritic cell (Fig. 6A). Then we excavated the correlations between the expression of SAA1 and immune cell infiltrations in the same way. Surprisingly, we found almost full agreement results (Fig. 6B).

### SAA1 promotes ccRCC cell migration via ERK-AP1-MMPs axis

**Figure 7:**
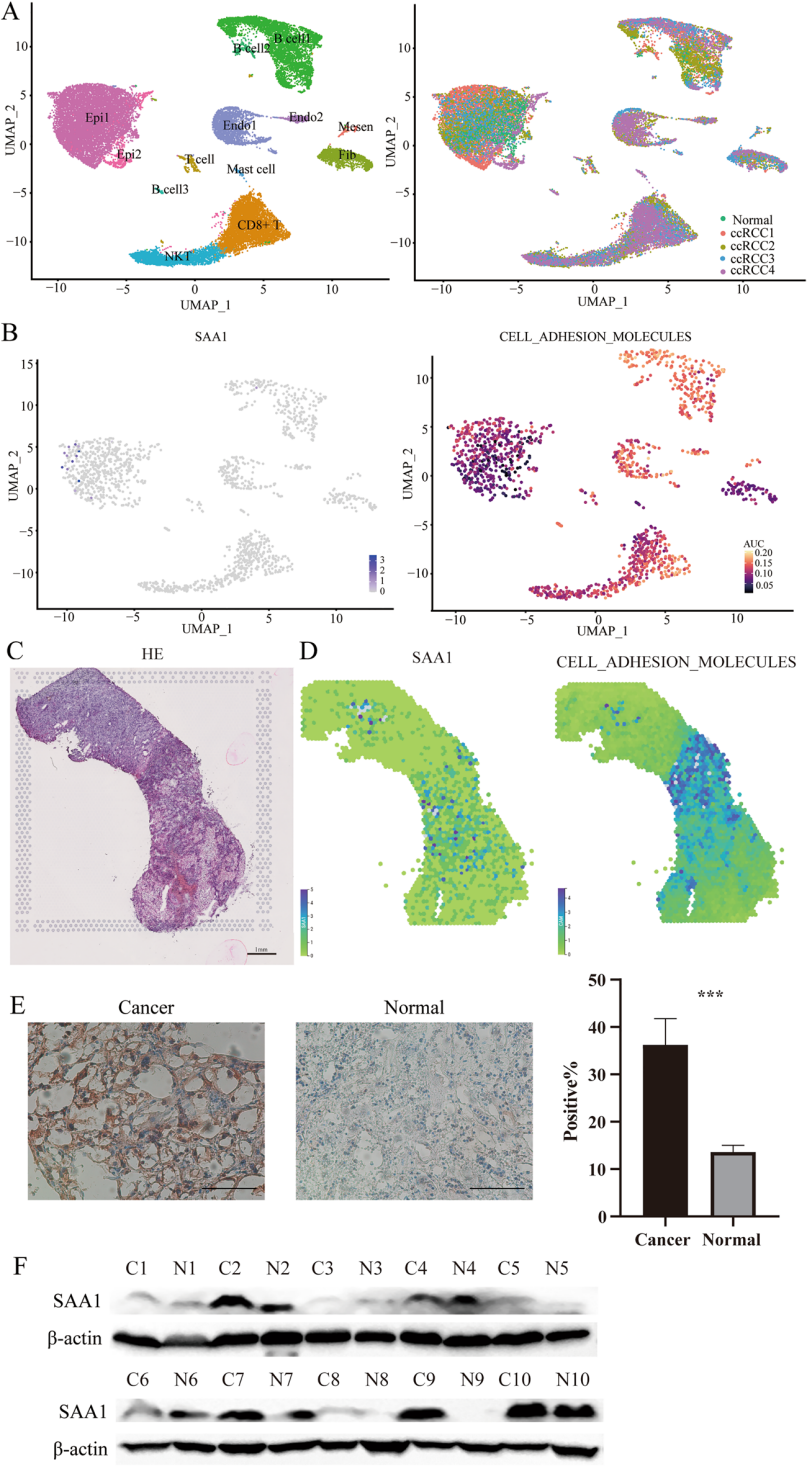
Expression status of SAA1 in ccRCC. (**A**) The distribution and annotations of single cells labelled by cluster identity (left panel) and tissue of origin (right panel) with UMAP. (**B**) Expression profiles of SAA1 (left panel) and cell adhesion molecules pathway score (right panel) for each cell. (**C**) HE staining of ccRCC tissues corresponding to the spatial transcriptomics results. (**D**) Expression profiles of SAA1 and cell adhesion molecules pathway score for each cell based the spatial transcriptome sequencing data. (**E**) Representative images of SAA1 immunohistochemistry and corresponding quantification. (**F**) The expression of SAA1 between ccRCC tissues and normal tissues (10 pairs).

In order to verify our previous results, was performed single-cell sequencing analysis and spatial transcriptome analysis. Expression profiles were further confirmed by analyzing the scRNA-seq data from GSE125938, which included one healthy kidney sample and two ccRCC samples, as well as GSE171306, which included two ccRCC samples. To elaborate, a grand total of 12 distinct cellular clusters were detected (Fig. 7A). Expression of SAA1 and cell adhesion molecules pathway score were visualized via UMAP (Fig. 7B). The results showed that, regions of high SAA1 expression highly overlapped with regions of cell adhesion molecules pathway hyperactivity in the tumor cell cluster. Similar results were found in spatial transcriptome analysis (Fig. 7C,D). Furthermore, immunohistochemistry confirmed the increased presence of SAA1 in patient samples (Fig. 7E). Western blotting results of paired samples from 10 pairs of ccRCC patients similarly demonstrated that SAA1 is highly expressed in tumor tissue (Fig. 7F).

**Figure 8:**
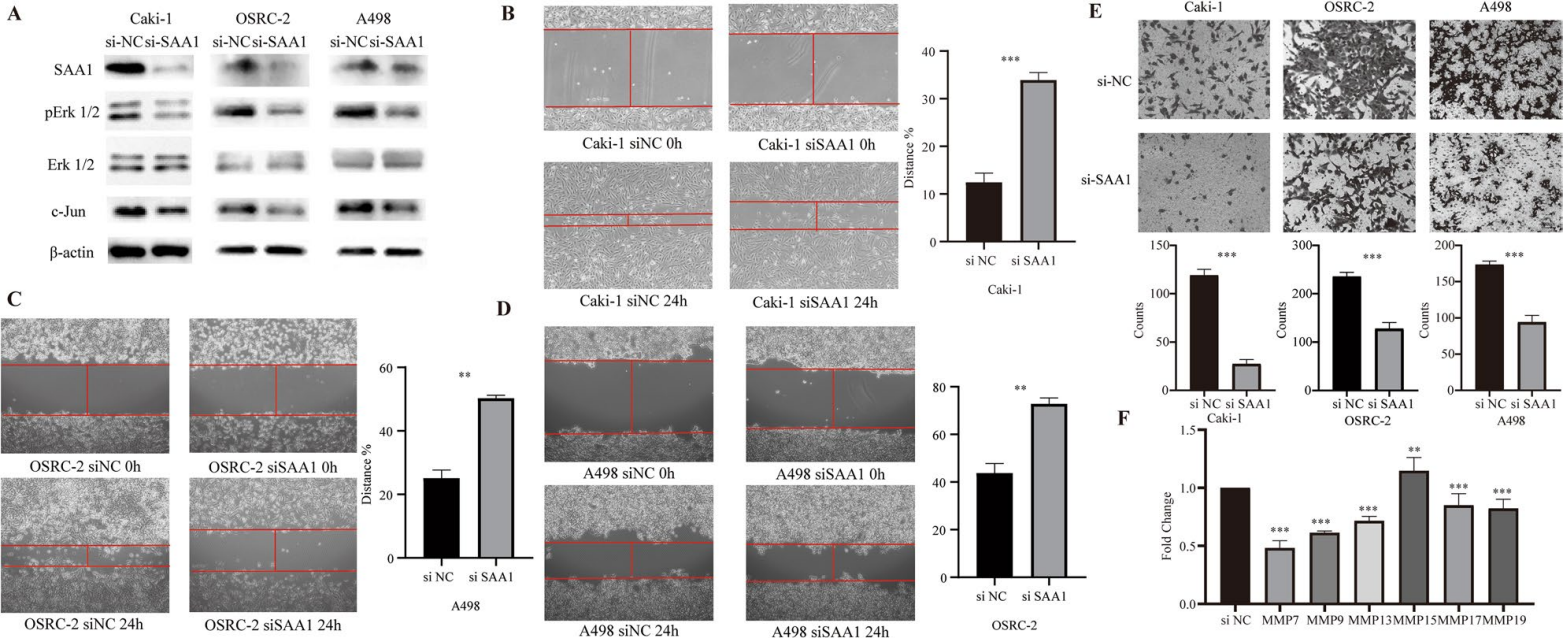
SAA1 promotes ccRCC cell migration via ERK-AP1-MMPs axis. (**A**) Expression of SAA1, ERK1/2, phosphorylated ERK1/2/, c-Jun, and β-actin detected after transfection with siRNA in Caki-1, OSRC-2 and A498 cell lines using western blotting. (**B**–**D**) The wound-healing assay and quantification of wound-healing assay data. (**E**) The transwell assay and quantification of transwell migration assay data. (**F**) RT-qPCR analysis of key MMPS marker (MMP7, MMP9, MMP13, MMP15, MMP17, MMP19).

Afterwards, we validated our conjecture by conducting in vitro experiments. In the Caki-1, OSRC-2 and A498 cells, we employed siRNAs that targeted SAA1 to suppress its expression. Confirmation of SAA1 knockdown was validated through western blotting (Fig. 8A and Supplementary Fig. 5A). The migration ability of Caki-1, OSRC-2 and A498 cells was hindered by the suppression of SAA1, as evidenced by the outcomes of wound-healing and transwell assays (Fig. 8B–E). The expression of ERK1/2, phosphorylated ERK1/2, and c-Jun was determined through western blotting (Fig. 8A). The expression level of c-Jun and the phosphorylation level of ERK1/2 were observed to be diminished to varying extents subsequent to the knockdown of SAA1.

Considering prior literature, c-Jun has been identified as a crucial transcription factor that facilitates the migration of tumor cells. Consequently, we hypothesized the potential regulatory role of c-Jun in the gene expression of core MMPs, which constitute prognostic models, utilizing the JASPAR database. We found that the protein expression level of c-Jun and the phosphorylation level of ERK1/2 in cells were also reduced to different degrees after knocking down SAA1. Based on previous reports in the literature, c-Jun is one of the important transcription factors that promote tumor cell migration. Therefore, we predicted whether c-Jun could regulate the gene expression of core MMPs, which is consist of prognostic models, through the JASPAR database (Supplementary Fig. 5B,C). It was surprising to discover that c-Jun possesses potential binding sites within the promoter regions of MMP7, MMP9, MMP13, MMP17, and MMP19, implying that c-Jun may exert transcriptional regulation over these core MMPs. Subsequently, we conducted RT-qPCR to assess the mRNA expression levels of core MMPs in Caki-1 cells with SAA1 knockdown (Fig. 8F). The findings demonstrated that the reduction in SAA1 and c-Jun expression levels corresponded to varying degrees of decrease in the mRNA expression levels of MMP7, MMP9, MMP13, MMP17, and MMP19.

## Discussion

The MMP family has been widely studied as the matrixin subfamily of the zinc metalloprotease family due to its ability to degrade the ECM^9^. Because of this, their actions are vital in the migration and metastasis of tumors. This function of the MMP family has been explored in several studies^25–31^. The link between the MMP family and ccRCC patients is also constantly mentioned. However, most studies have focused on MMP2 and MMP9, and several studies have shown MMP9 and MMP2 were associated with the invasion and metastasis of ccRCC^18,32^. MMP2 and MMP9 also have been identified as significant contributors to the process of epithelial-to-mesenchymal transition (EMT) in ccRCC cells^33^. Previous research has demonstrated that the activation of MMP2 in ccRCC can lead to the progression of EMT^34^. Additionally, MMP-7 is regarded as a standalone predictive element for patients with ccRCC^35^. In light of these findings, a comprehensive analysis was conducted in the context of pan-cancer, which yielded significant findings regarding the variations observed in the characteristics of MMPs across various carcinomas. Particularly, MMPs exhibited distinct prognostic value in ccRCC. This observation aligns with the conclusions of a previous pan-cancer study^11^. Consequently, we selected ccRCC as the focus of our further investigation.

Subsequently, we introduced molecular typing utilizing 23 MMPs to discern ccRCC patients, thereby undertaking an initial investigation into its clinical significance. Through this process, two distinct clusters were discerned, with cluster 1 exhibiting a high expression of most MMPs. Further examination of subcluster characteristics revealed a correlation between cluster 1 patients and higher clinical stage, pathological grade, and unfavorable prognosis. These findings align with the prevailing body of literature, which posits a pro-cancer role for MMPs in ccRCC^36,37^. The findings from the enrichment analysis also indicate a significant correlation between cell adhesion and the unfavorable prognosis of cluster 1 patients. Given the significance of immunotherapy in renal cancer treatment, we proceeded to examine variations in immunotherapy-related indicators across different subclusters. Remarkably, our investigation revealed a notable elevation in M2 macrophage infiltration levels among patients in cluster 1. M2 macrophages are recognized for their significant contribution to the establishment of an immunosuppressive microenvironment through their facilitation of tumor cell migration and evasion of immune surveillance^38,39^. Meanwhile, there was a notable increase in the expression levels of PDCD1LG2 and SIGLEC15 in cluster 1 patients. The prediction results based on the TIDE algorithm indicated that cluster 1 patients possessed a comparatively robust ability to evade immune responses. PDCD1LG2 and SIGLEC15, as emerging targets for immunotherapy, hold considerable promise for patients resistant to anti-PDL1 treatment^40,41^. Our findings imply that conventional immunotherapy protocols may have limited efficacy in patients with heightened MMP activity, whereas targeting PDCD1LG2 and SIGLEC15 may yield more favorable outcomes. Finally, we conducted an assessment of variations in drug sensitivity among distinct subclusters. Notably, Cluster 1 demonstrated heightened sensitivity towards a majority of targeted drugs and chemotherapeutic agents. Conversely, Cluster 2 exclusively exhibited sensitivity towards lapatinib. It is worth mentioning that previous investigations on lapatinib's efficacy in treating ccRCC patients have yielded unfavorable outcomes, with only a subset of individuals potentially benefiting from its administration^42^. The findings from our study hold potential implications for the implementation of personalized treatment strategies for ccRCC patients.

We performed Lasso regression to construct an MMP-related prognostic risk model in the TCGA-KIRC cohort. The robustness and accuracy of the risk model were validated in the E-MATB-1980 cohort and different subgroups of the TCGA-KIRC cohort. We conducted additional investigations into the potential mechanism driven by MMPs to facilitate the advancement of ccRCC. Among the high-risk group, SAA1 exhibited the most notable upregulation. The SAA1-H MMPS-H subgroup demonstrated the poorest OS prognosis, indicating a strong association between SAA1 and MMPs. This perspective was further supported by the outcomes of the enrichment analysis. Enrichment analysis results of DEGs between the high-risk group and the low-risk group and enrichment analysis results of SAA1-related genes showed a high level of similarity. Cell adhesion/migration-related pathways and immune-related pathways were significantly enriched in both gene sets. Previous studies have also identified the close association between SAA1 and tumor metastasis^43,44^. Nevertheless, no study has yet elucidated the plausible regulatory connection between MMP and SAA1 in ccRCC. Additionally, the enrichment analysis has demonstrated noteworthy associations between genes related to SAA1 and the ERK and AP-1 signaling pathways, suggesting that SAA1 may regulate the expression of MMPs via modulation of the ERK-AP1 axis. Considering that a high proportion of the enrichment analysis results were associated with immune-related pathways, we further evaluated the relationship between immune cell infiltration and risk scores. Risk scores were indeed significantly correlated with most immune cell infiltrations. Surprisingly, the relationship between immune cell infiltration and SAA1 expression showed similar patterns.

Lastly, we conducted additional experiments to partially confirm the findings in ccRCC. Initially, we confirmed the expression of pivotal genes at the single-cell level, spatial transcriptome level and tissue level. We found that cells with higher SAA1 expression also had higher activation of cell adhesion molecules pathway. Subsequently, we assessed the alterations in cellular migration capabilities, along with the potential downstream effects on ERK-AP1-MMPs by knocking down SAA1 in the cell lines. Our findings indicate that SAA1 modulates the migration ability of tumor cells via the ERK-AP1-MMPs axis.

Nonetheless, there are several drawbacks to our investigation. Although we verified the results via multiple methods, more data is required. In vivo experiments were lacking to validate these results. Despite these constraints, we managed to acquire somewhat dependable findings, and we intend to further substantiate the existing outcomes and investigate potential mechanisms in future research.

## Methods

### Data access

RNA-seq expression data and corresponding clinical follow-up information of The Cancer Genome Atlas (TCGA)—kidney renal clear cell carcinoma (KIRC) cohort and the E-MTAB-1980 cohort were acquired from the Genomic Data Commons (GDC) data portal of the TCGA database and the ArrayExpress database respectively. Pan-cancer genomic and transcriptomic data were also downloaded from the TCGA database, and corresponding follow-up information was collected, following a methodology similar to previous reports^45,46^. The TCGA-KIRC cohort contained 532 ccRCC samples and 161 normal samples, while the E-MTAB-1980 cohort contained 101 ccRCC samples. The number of patients involved in other cohorts are summarized in Supplementary Table 1. The GSE152938^47^ and GSE171306^48^ dataset was obtained from the Gene Expression Omnibus (GEO) dataset. Single-cell sequencing data of 2 ccRCC samples and 1 normal kidney sample from GSE152938 and 2 ccRCC samples from GSE171306 were used in this study. The spatial transcriptomic data used in this study is available at the European Genome-Phenome Archive: EGAD00001008781^49^. The spatial transcriptomic data analysis was performed by the online web portal provided by Wellcome Sanger Institute (https://www.sanger.ac.uk/).

### Pan-cancer analysis

Initially, the fold change (FC) value was computed to evaluate the changes in gene expression between tumor tissues and normal tissues. Then, the univariate Cox regression analysis was performed to identify the prognostic value of each gene. Subsequently, the CNV, both amplified and deleted, was tallied. In the case of SNV, the mutation frequency (number of samples with SNV divided by the total number of samples) was computed. The outcomes were visualized using a heatmap presentation with the ggplot2 package. Survival Prognosis analysis was performed through “forestplot”, “survival” and “survminer” packages.

### Molecular subtyping identification

In order to characterize the expression patterns of the MMP family in ccRCC, a method similar to previous research was used to identity the distinct molecular subtypes. We utilized the “ConsensusClusterPlus” package for consensus clustering analysis, “survival” package for survival analysis, “Limma” package for differential expression analysis, and “ClusterProfiler” package for enrichment analysis.

### Immune related analysis and therapeutic sensitivity analysis

The immune microenvironment of different molecular subtypes was explored through analysis of immune cell infiltration and immune checkpoint expression levels. Multiple algorithms including XCELL, CIBERSORT, EPIC, and TIMER were utilized to predict immune cell infiltration abundance in the TCGA-KIRC cohort. The TIDE algorithm was utilized to predict immune checkpoint inhibitor responsiveness. The drug sensitivity in different KIRC subtypes was investigated by “oncoPredict”. Correlation heatmaps, bubble plots and scatter plots were plotted using the ggplot2 package. Spearman’s correlation was used to analyze the correlation. The Wilcoxon test was used to evaluate statistical differences.

### Prognostic risk model development and validation

In the TCGA-KIRC cohort, the development of the MMPs-related prognostic risk model was carried out using the LASSO regression algorithm and Cox regression analysis. This model was then validated in the E-MTAB-1980 cohort. Based on the median riskscore value in the training set, patients were categorized into high-risk and low-risk groups. Similar approaches were applied to the test set. The R package “glmnet” was used to perform LASSO regression algorithm, while “survival” was used for Kaplan–Meier (KM) analysis. The time-dependent receiver-operating characteristic (ROC) curves were used to compare the predictive accuracy of the risk score.

### GeneMANIA database

GeneMANIA is a user-friendly network mapping tool. Through the utilization of this database, we conducted an analysis to map the potential network of interactions associated with SAA1, subsequently visualizing the results using the Cytoscape software^50^.

### Single cell RNA-seq data analysis

Following a previously described method, scRNA-Seq data were processed^51^. The cellMarker database was used to manually annotate cell clusters^52^. UMAP was used to perform dimensional reduction and visualize cluster classifications. The analysis was performed through “Seurat”, “Harmony” and “AUCell” R packages.

### Transcription factor binding site analysis

The 2000 bp upstream regions of genes were chosen as potential promoter sequences. These potential sequences, where promoters are situated, were acquired from the National Center of Biotechnology Information (NCBI). The JASPAR software was employed to predict transcription factor binding sites. The binding likelihood (relative score) was established at 0.9.

### Specimen collection

From June to September 2023, we gathered 10 pairs of ccRCC and corresponding adjacent tissues from patients who underwent partial or radical nephrectomy at the First Hospital of Dalian Medical University (Dalian, China).

The Ethics Committee of the Second Hospital of Tianjin Medical University granted its approval to the patient sample research (KY2023K118). All experiments were performed in accordance with relevant guidelines and regulations. Every participant granted their written consent after receiving appropriate information.

### Real-time quantitative PCR (RT-qPCR)

RT-qPCR was employed to detect the relative mRNA expression, utilizing the fast start universal SYBR green master kits (S2008, BR Healthcare, China) and real-time PCR System (CFX96TM, Bio-Rad, USA). Supplementary Table 2 contains a comprehensive list of all the primers.

### Cell culture and antibody

Tianjin Institute of Urology (Tianjin, China) provided the Caki-1, A498 and OSRC-2 cell lines. ENCODE cell culture standards were followed when growing the cells.

The primary anti-SAA1 (A1655, ABclonal, China), anti-ERK1/2 (A4782, ABclonal, China), anti- Phospho-ERK1-T202/Y204 + ERK2-T185/Y187 (AP0974, ABclonal, China), anti-c-Jun (A0246, ABclonal, China), and anti-ß-Actin (AC026, ABclonal, China) antibodies were used according to the instructions.

### Western blotting

To extract total proteins, RIPA buffer was used. The proteins were separated on 10% or 15% resolving gels before transfer onto NC membranes with SDS-PAGE. After overnight incubation with diluted primary antibodies, membranes were incubated with secondary antibodies for 30 min.

### Immunohistochemical analysis

Immunohistochemistry was performed using unstained slides prepared from tumor tissue paraffin blocks. In summary, paraffin sections underwent immunostaining using a streptavidin peroxidase technique following antigen heat repair. The detection of the signal was accomplished by utilizing a solution of 3,3′-diaminobenzidine and hematoxylin.

### siRNA and transfection

JiKai Gene (Shanghai, China) provided the SiRNA targeting SAA1 and its respective control, which were introduced into cells using Lipofectamine 6000 reagent (C0526, Beyotime).

### Wound-healing assay

A total of 200,000 cells were seeded in 6-well dishes and allowed to grow until reaching 90% confluence. Next, a sterile pipette tip with a volume of 1000 μL was employed to gently scrape the fully-grown cell layer, resulting in the formation of a straight wound. Regular observations and imaging were conducted on the wound after a duration of 24 h.

### Transwell assay

In the upper chamber of 24-well plates (Corning, 3422, USA), we added 20,000 cells along with 200 µl of serum-free medium, while in the lower chamber, we added 600 μl complete medium. Following a 16-h incubation period, the cells were treated with a 1% solution of crystal violet for a duration of 5 min subsequent to fixation with a 4% tissue fixative for 20 min. Finally, the relocated cells were examined under a microscope.

### Statistical analysis

Both SPSS 23 and R software v4.03 were utilized in the analyses of statistical data. The gene expression levels, risk scores, and the abundance of immune cell infiltration between groups were analyzed via the Wilcoxon test. The correlation was analyzed by Spearman's correlation test. *P*-values of less than 0.05 indicated a significance level.

### Supplementary Information

Supplementary Information.

Supplementary Figures.

Supplementary Tables.

## Data Availability

The datasets analysed during the current study are available in the Cancer Genome Atlas database (https://portal.gdc.cancer.gov/) and the ArrayExpress database (https://www.ebi.ac.uk/biostudies/arrayexpress). The GSE152938 and GSE171306 dataset was obtained from the Gene Expression Omnibus dataset. The authors claim that all data describing the results of the study can be found in the text of the article or in Supporting information documents.
