# Supplementary material for: MMPs-related risk model identification and SAA1 promotes clear cell renal cell carcinoma migration via ERK-AP1-MMPs axis
Source: Sci Rep. 2024 Apr 24;14:9411. doi: 10.1038/s41598-024-59112-5 (PMC11043417; doi:10.1038/s41598-024-59112-5)

1.The original data for Figure 8A

1.1 B-actin for Caki-1 cell line

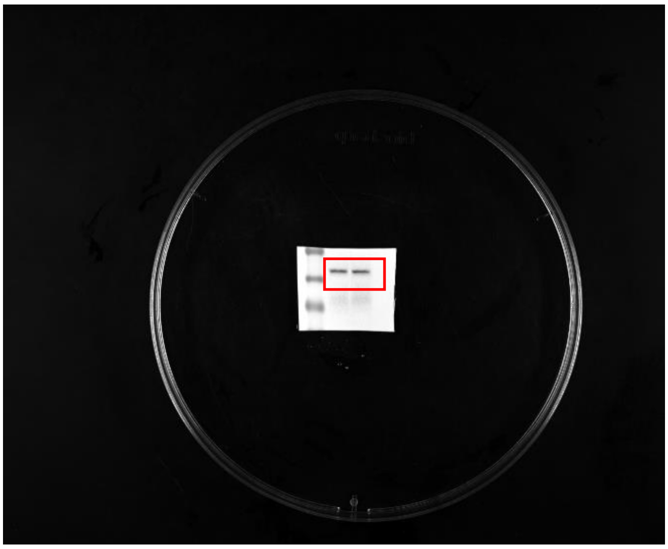

1.2 SAA1 for Caki-1 cell line

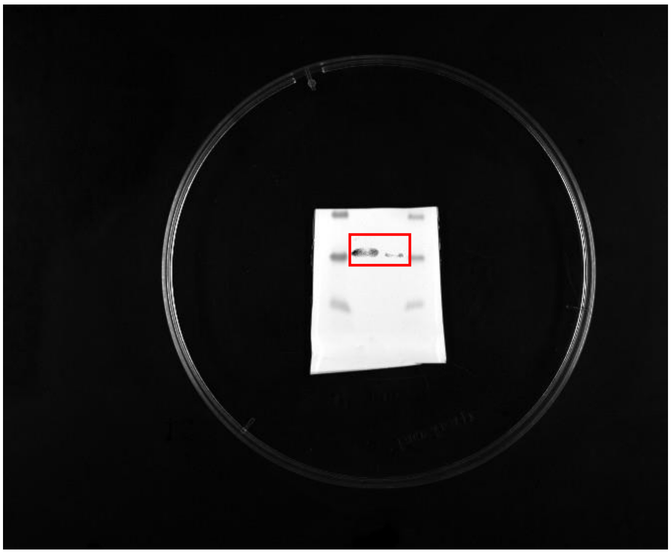

1.3 c-Jun for Caki-1 cell line

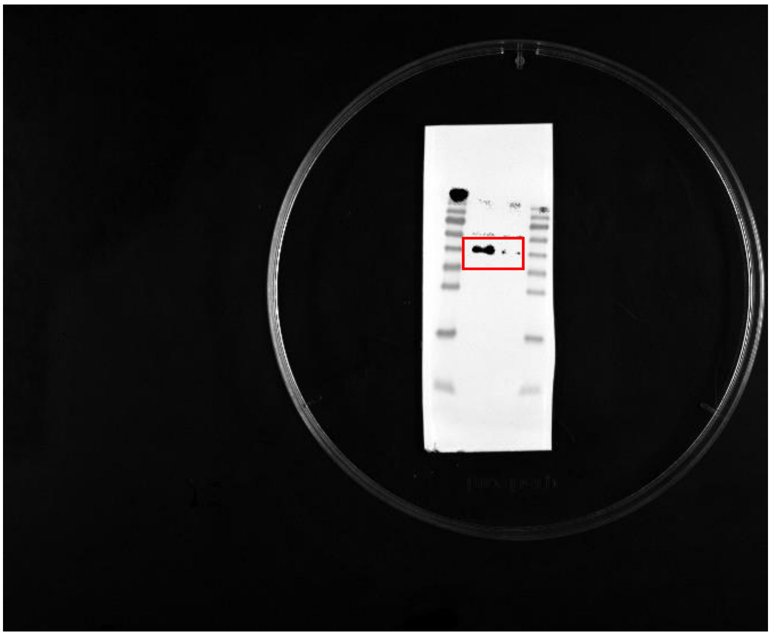

1.4 Erk 1/2 for Caki-1 cell line

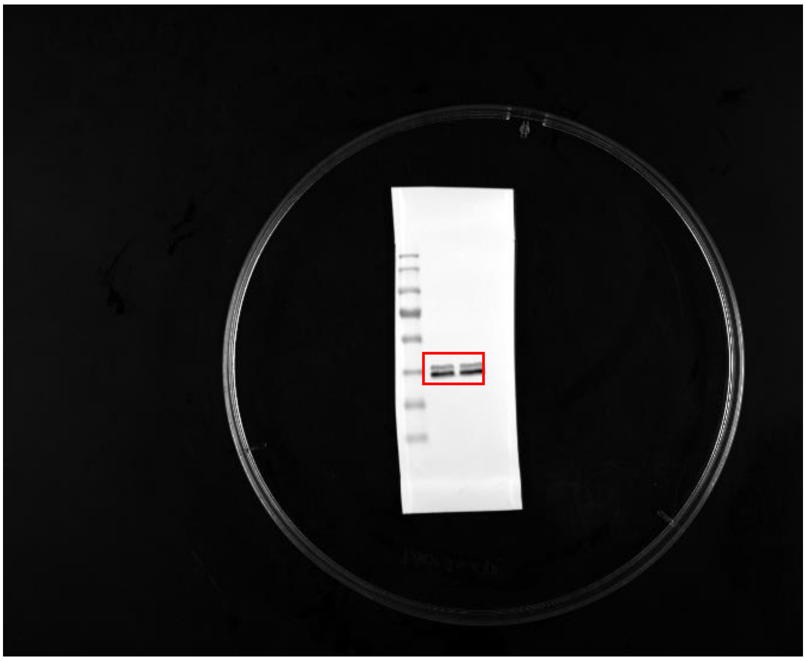

1.4 P-Erk 1/2 for Caki-1 cell line

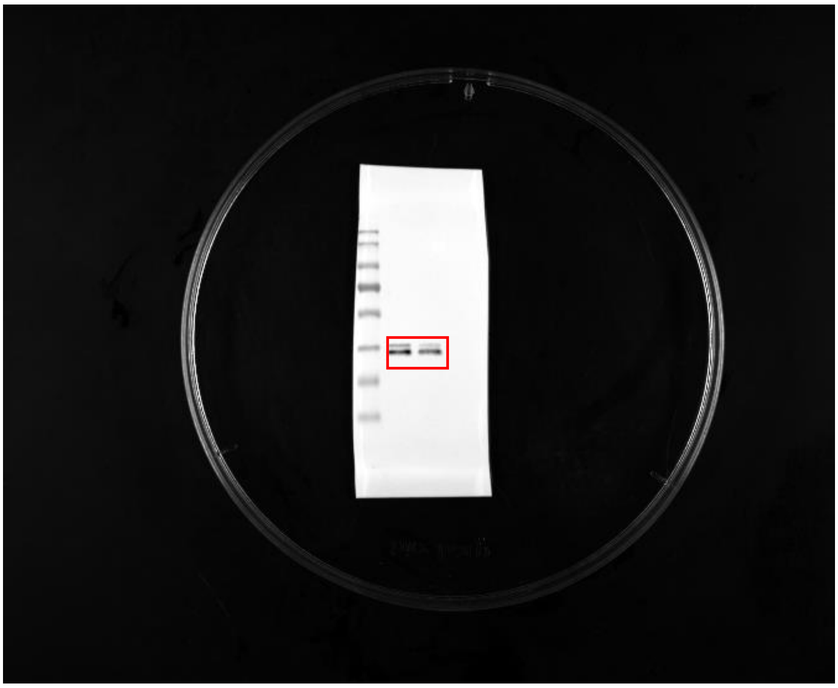

1.5 B-actin for OSRC-2 cell line

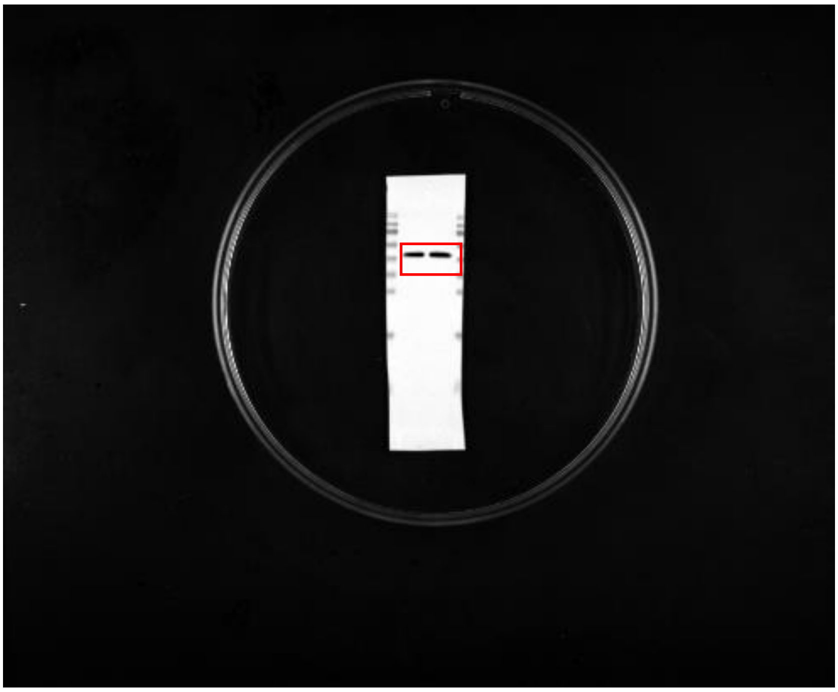

1.6 SAA1 for OSRC-2 cell line

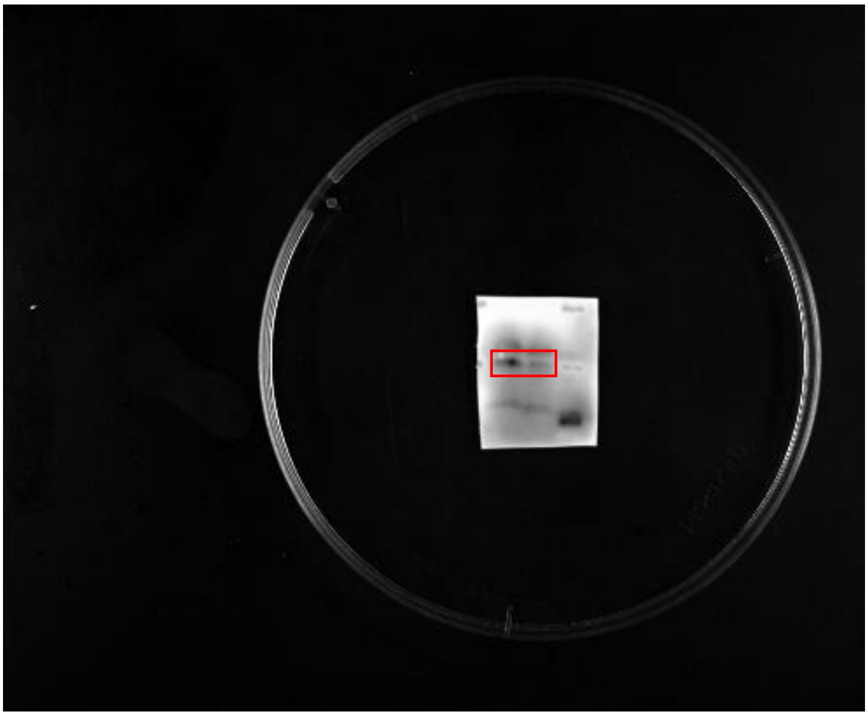

1.7 c-Jun for OSRC-2 cell line

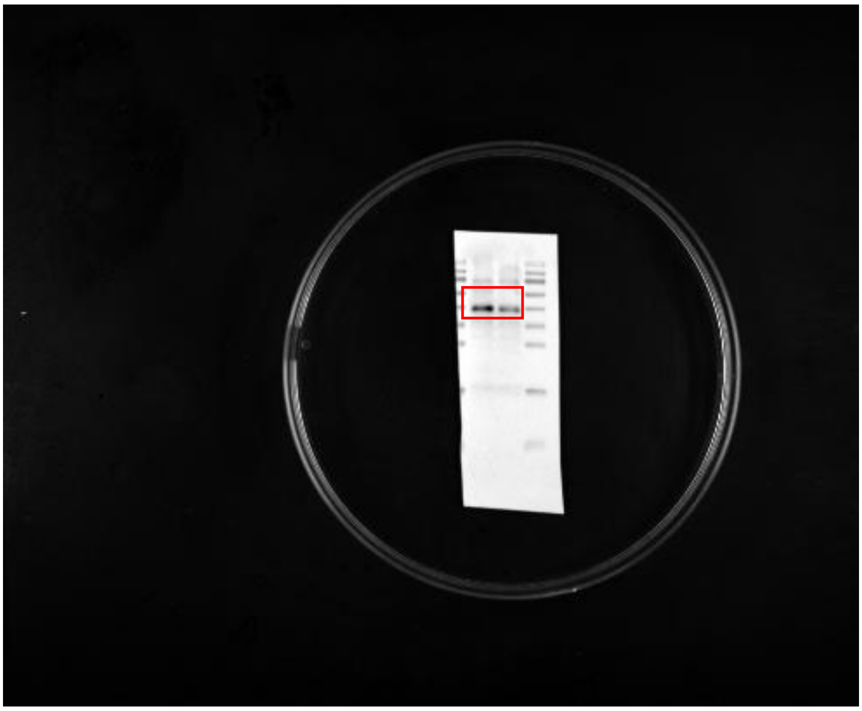

1.8 Erk1/2 for OSRC-2 cell line

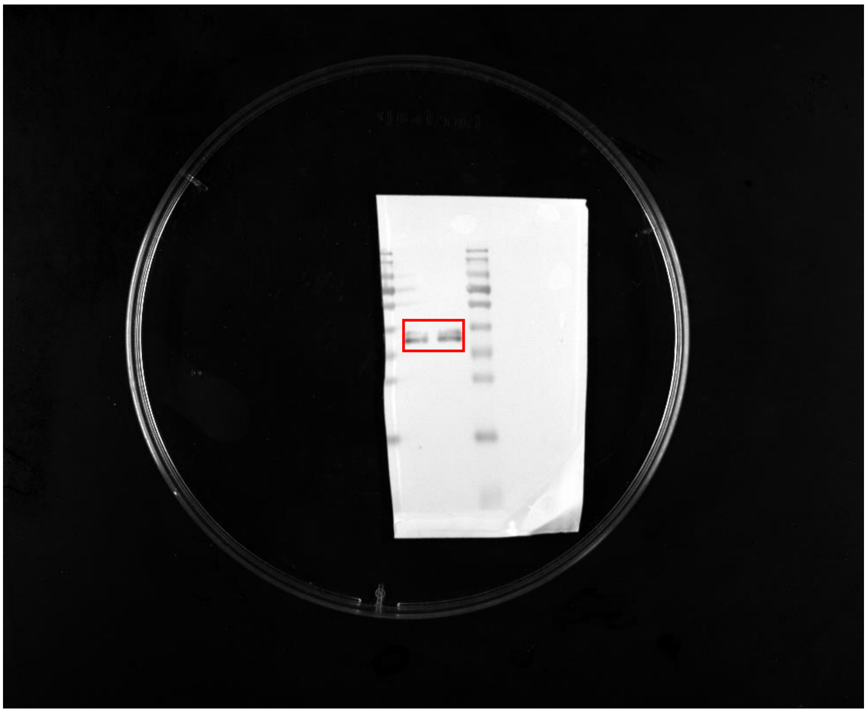

1.9 P-Erk1/2 for OSRC-2 cell line

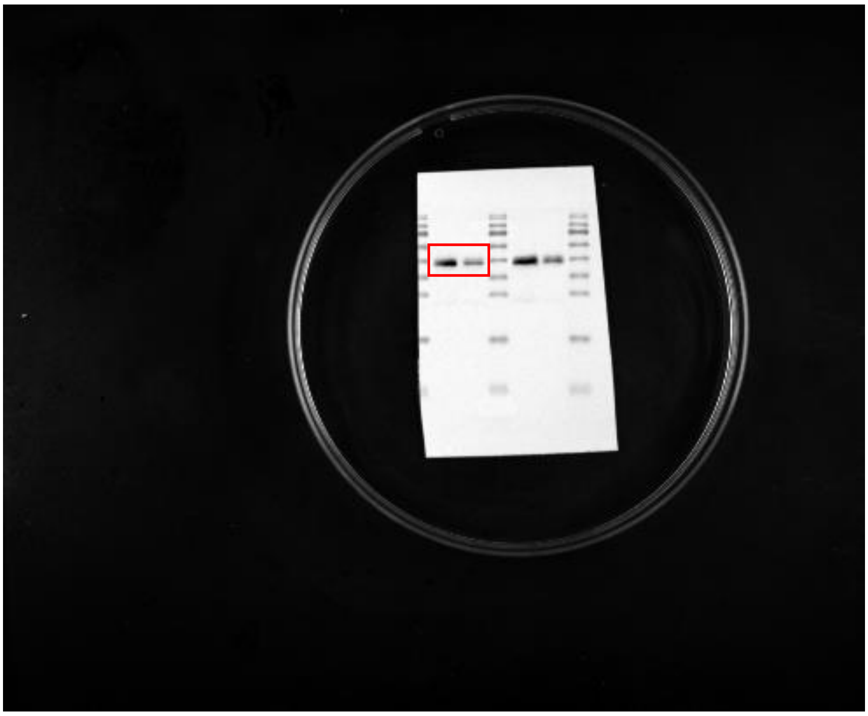

1.10 B-actin for A498 cell line

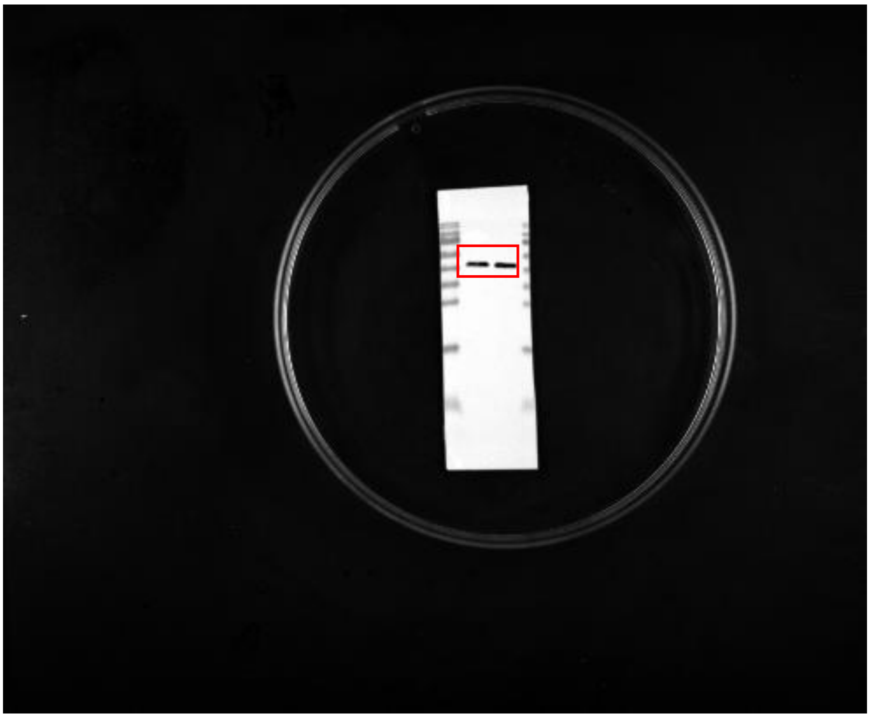

1.11 SAA1 for A498 cell line

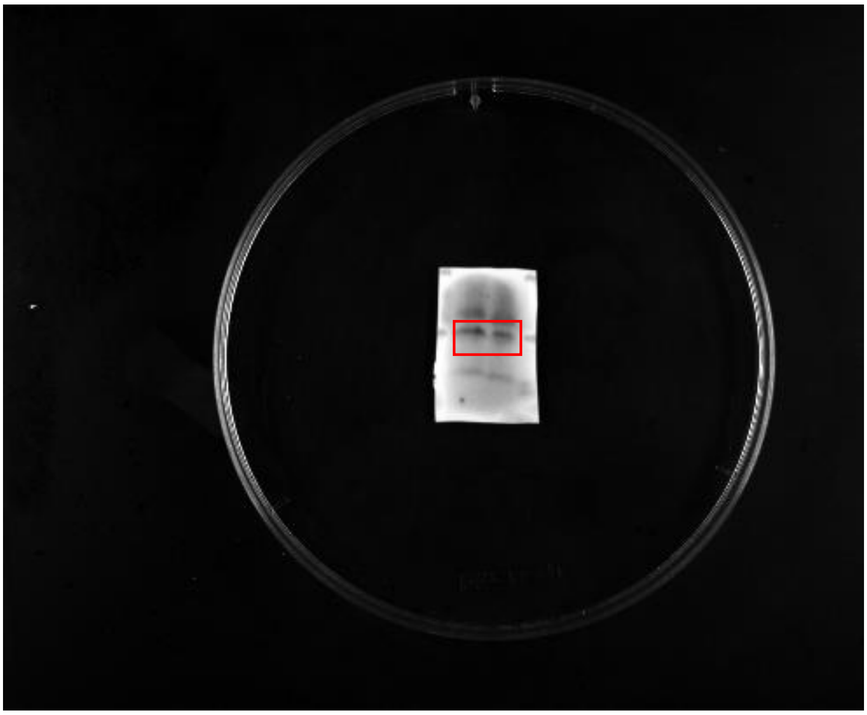

1.12 c-Jun for A498 cell line

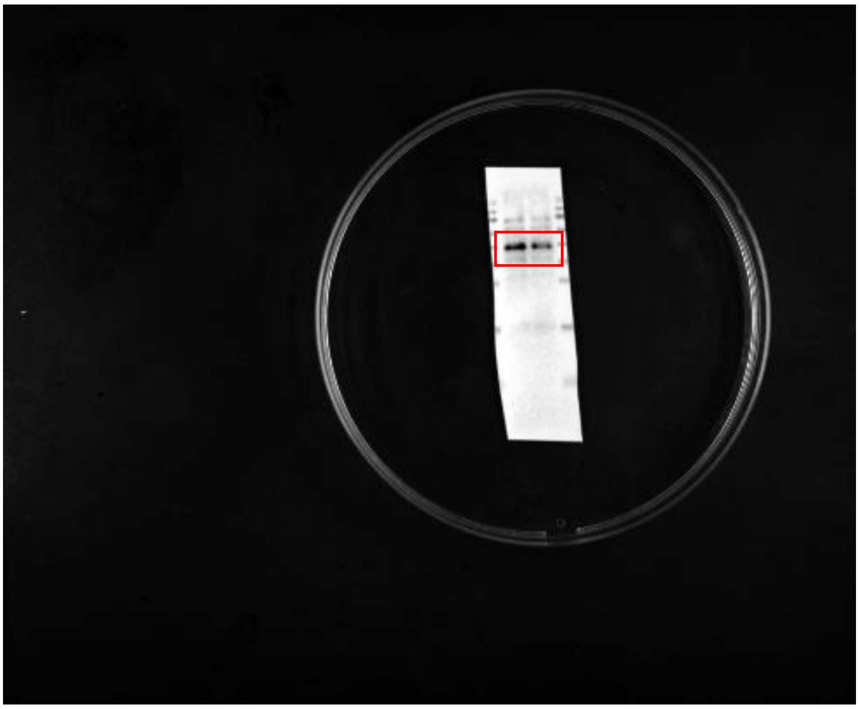

1.13 Erk1/2 for A498 cell line

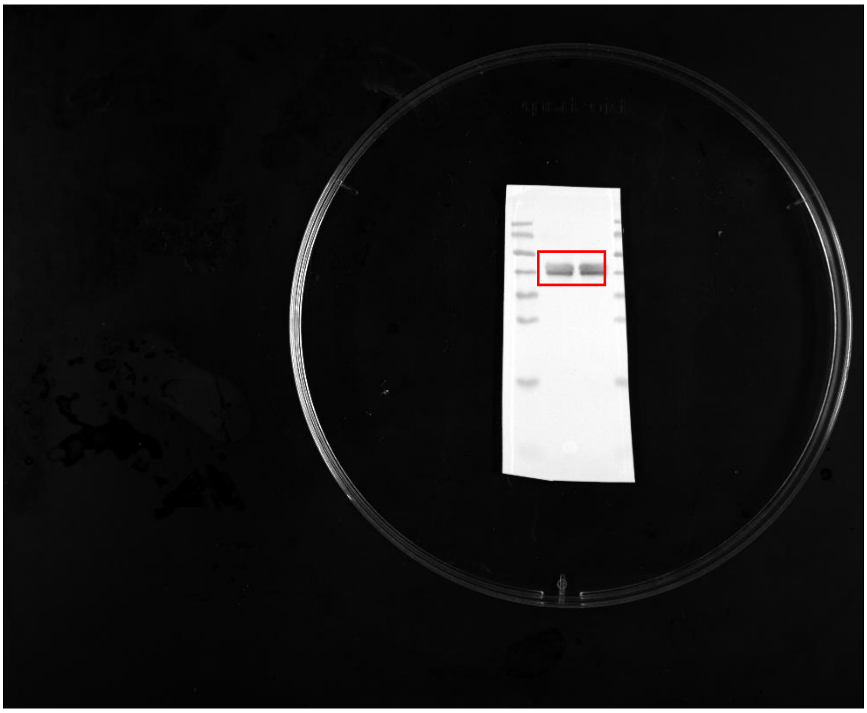

1.14 P-erk1/2 for A498 cell line

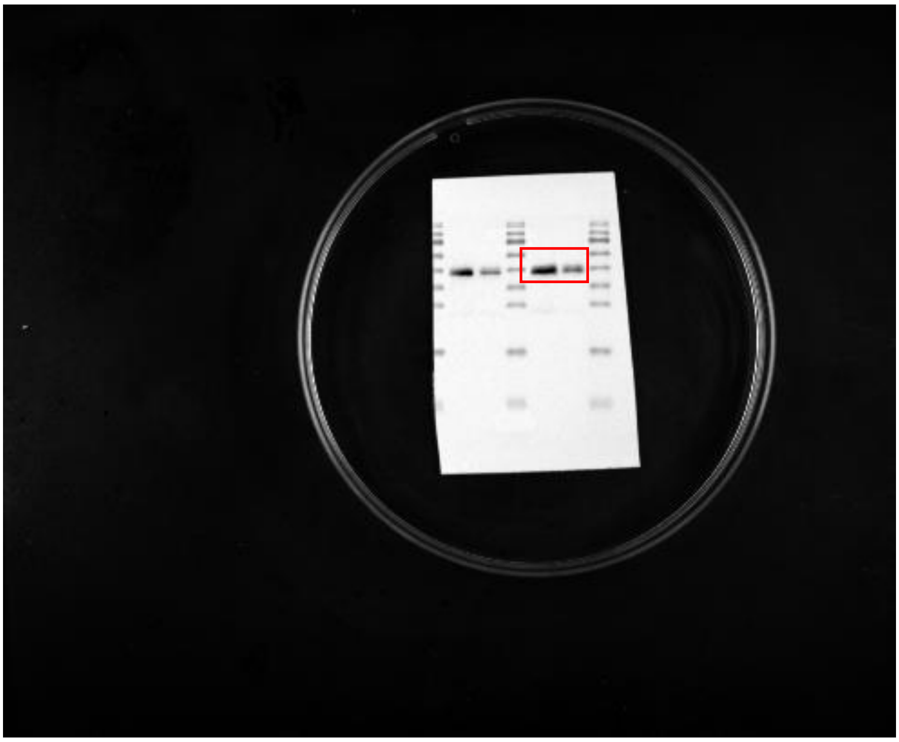

2.The original data for Figure 7F

2.1 b-actin

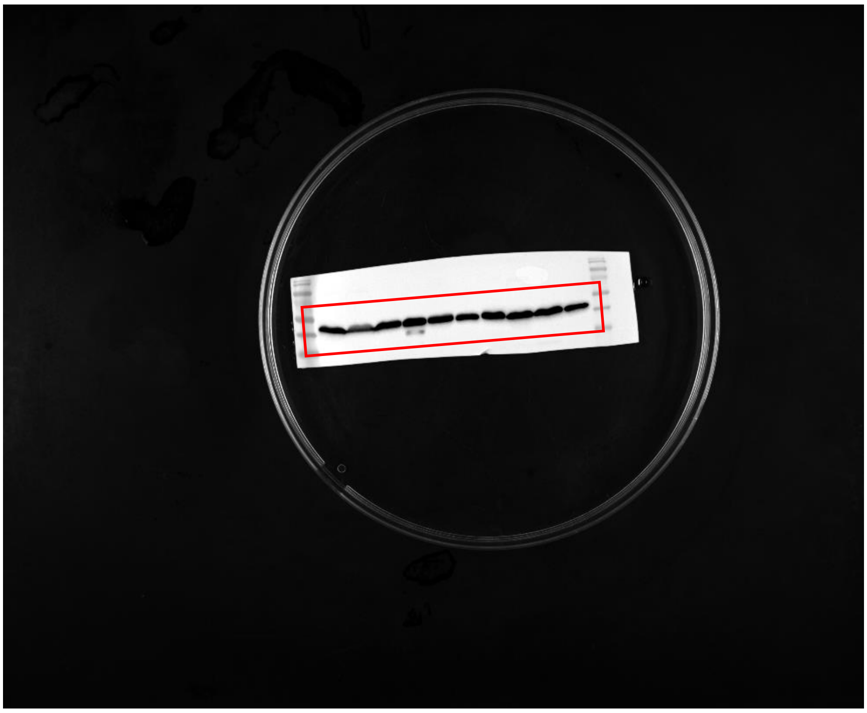

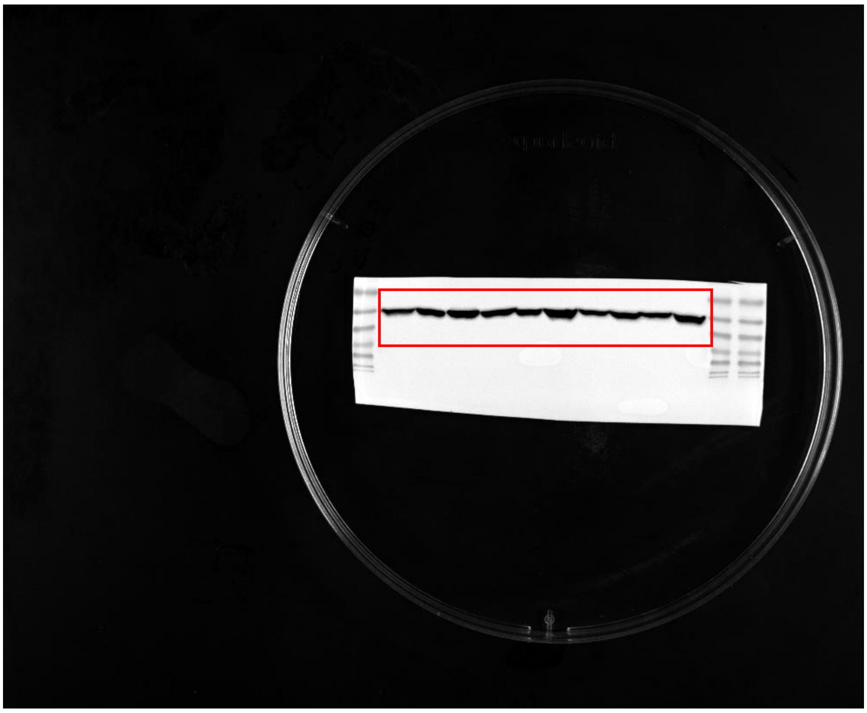

2.2 SAA1

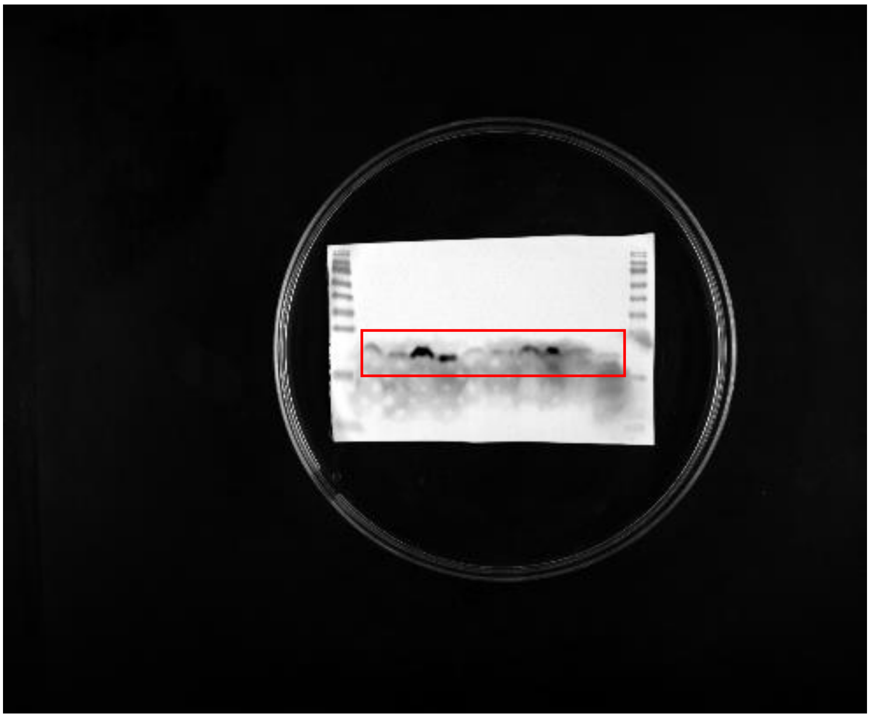

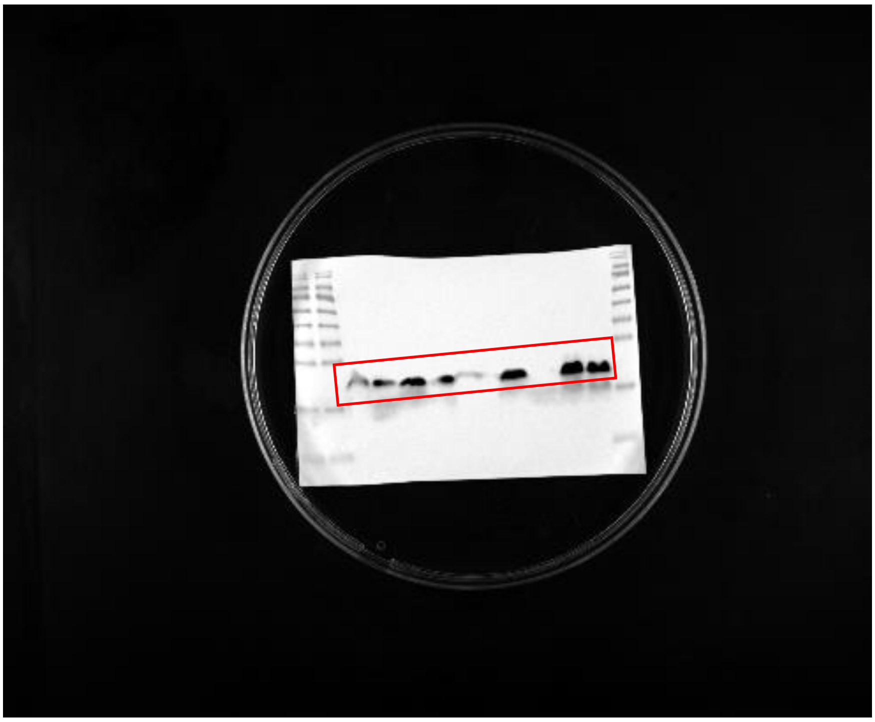

3.The original data for Supplementary Figure 5A

3.1 b-actin

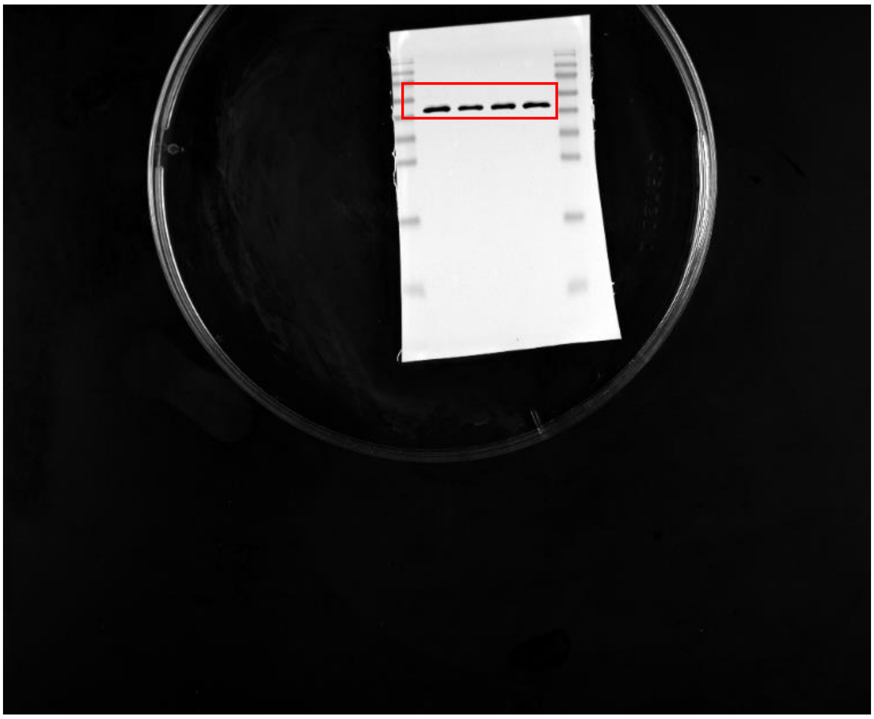

3.2 SAA1

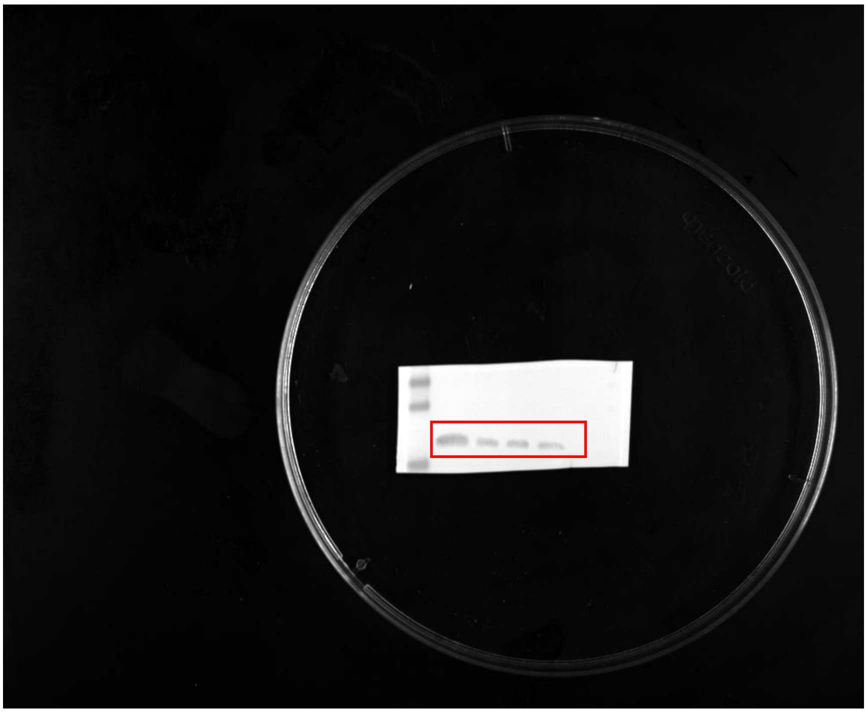

Supplement: Supplementary file 1 — Supplementary Information. [file 41598_2024_59112_MOESM1_ESM.pdf]
