# Supplementary material for: MMPs-related risk model identification and SAA1 promotes clear cell renal cell carcinoma migration via ERK-AP1-MMPs axis
Source: Sci Rep. 2024 Apr 24;14:9411. doi: 10.1038/s41598-024-59112-5 (PMC11043417; doi:10.1038/s41598-024-59112-5)
Supplement: Supplementary file 2 — Supplementary Figures. [file 41598_2024_59112_MOESM2_ESM.pdf]

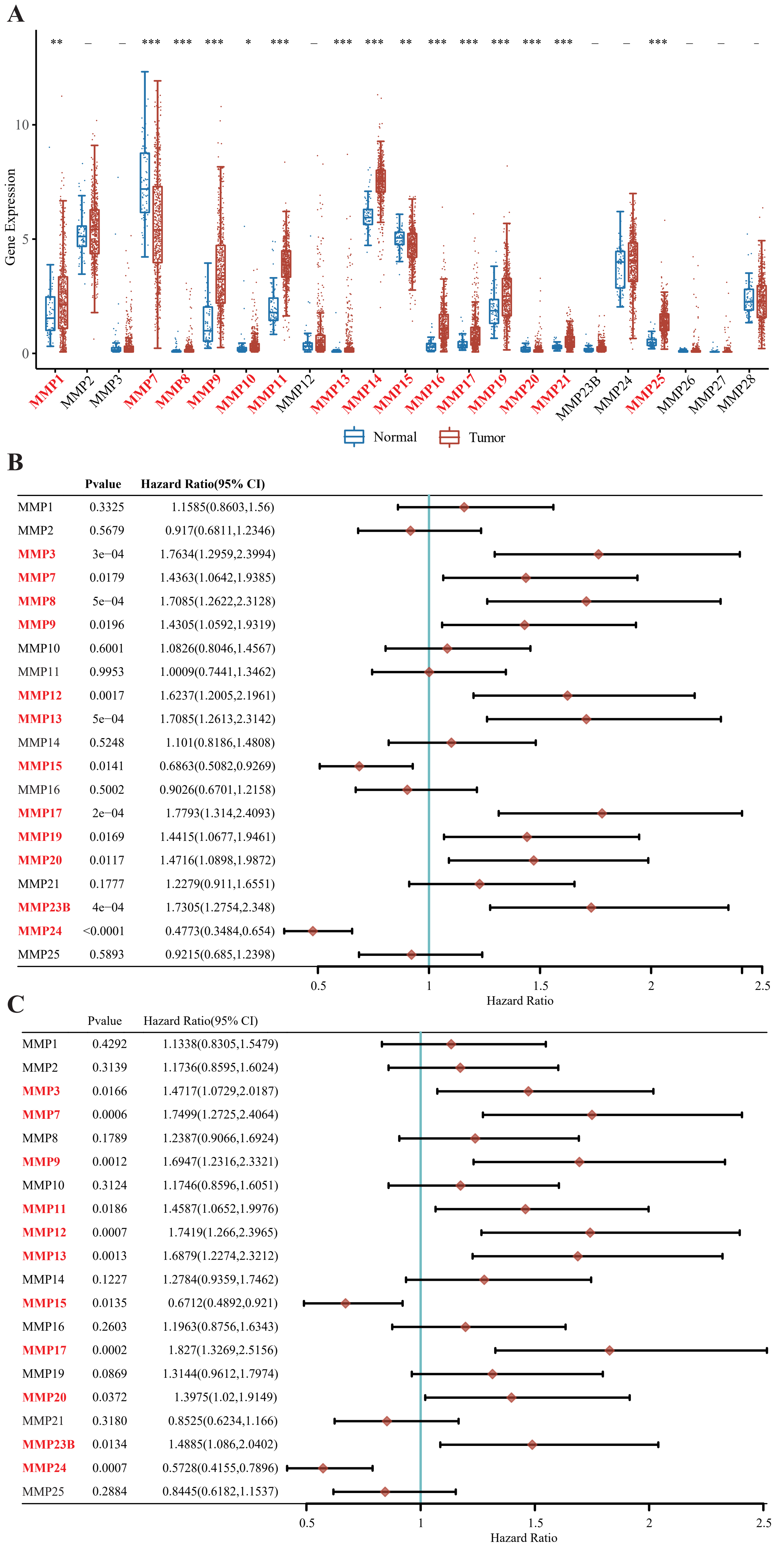
A
** – – *** *** *** * *** – *** *** ** *** *** *** *** *** – – *** – – –
Gene Expression
0
5
10
MMP1 MMP2 MMP3 MMP7 MMP8 MMP9 MMP10 MMP11 MMP12 MMP13 MMP14 MMP15 MMP16 MMP17 MMP19 MMP20 MMP21 MMP23B MMP24 MMP25 MMP26 MMP27 MMP28
Normal
Tumor
B
Pvalue Hazard Ratio(95% CI)
MMP1 0.3325 1.1585(0.8603,1.56)
MMP2 0.5679 0.917(0.6811,1.2346)
MMP3 3e−04 1.7634(1.2959,2.3994)
MMP7 0.0179 1.4363(1.0642,1.9385)
MMP8 5e−04 1.7085(1.2622,2.3128)
MMP9 0.0196 1.4305(1.0592,1.9319)
MMP10 0.6001 1.0826(0.8046,1.4567)
MMP11 0.9953 1.0009(0.7441,1.3462)
MMP12 0.0017 1.6237(1.2005,2.1961)
MMP13 5e−04 1.7085(1.2613,2.3142)
MMP14 0.5248 1.101(0.8186,1.4808)
MMP15 0.0141 0.6863(0.5082,0.9269)
MMP16 0.5002 0.9026(0.6701,1.2158)
MMP17 2e−04 1.7793(1.314,2.4093)
MMP19 0.0169 1.4415(1.0677,1.9461)
MMP20 0.0117 1.4716(1.0898,1.9872)
MMP21 0.1777 1.2279(0.911,1.6551)
MMP23B 4e−04 1.7305(1.2754,2.348)
MMP24 <0.0001 0.4773(0.3484,0.654)
MMP25 0.5893 0.9215(0.685,1.2398)
0.5 1 1.5 2 2.5
Hazard Ratio
C
Pvalue Hazard Ratio(95% CI)
MMP1 0.4292 1.1338(0.8305,1.5479)
MMP2 0.3139 1.1736(0.8595,1.6024)
MMP3 0.0166 1.4717(1.0729,2.0187)
MMP7 0.0006 1.7499(1.2725,2.4064)
MMP8 0.1789 1.2387(0.9066,1.6924)
MMP9 0.0012 1.6947(1.2316,2.3321)
MMP10 0.3124 1.1746(0.8596,1.6051)
MMP11 0.0186 1.4587(1.0652,1.9976)
MMP12 0.0007 1.7419(1.266,2.3965)
MMP13 0.0013 1.6879(1.2274,2.3212)
MMP14 0.1227 1.2784(0.9359,1.7462)
MMP15 0.0135 0.6712(0.4892,0.921)
MMP16 0.2603 1.1963(0.8756,1.6343)
MMP17 0.0002 1.827(1.3269,2.5156)
MMP19 0.0869 1.3144(0.9612,1.7974)
MMP20 0.0372 1.3975(1.02,1.9149)
MMP21 0.3180 0.8525(0.6234,1.166)
MMP23B 0.0134 1.4885(1.086,2.0402)
MMP24 0.0007 0.5728(0.4155,0.7896)
MMP25 0.2884 0.8445(0.6182,1.1537)
0.5 1 1.5 2 2.5
Hazard Ratio

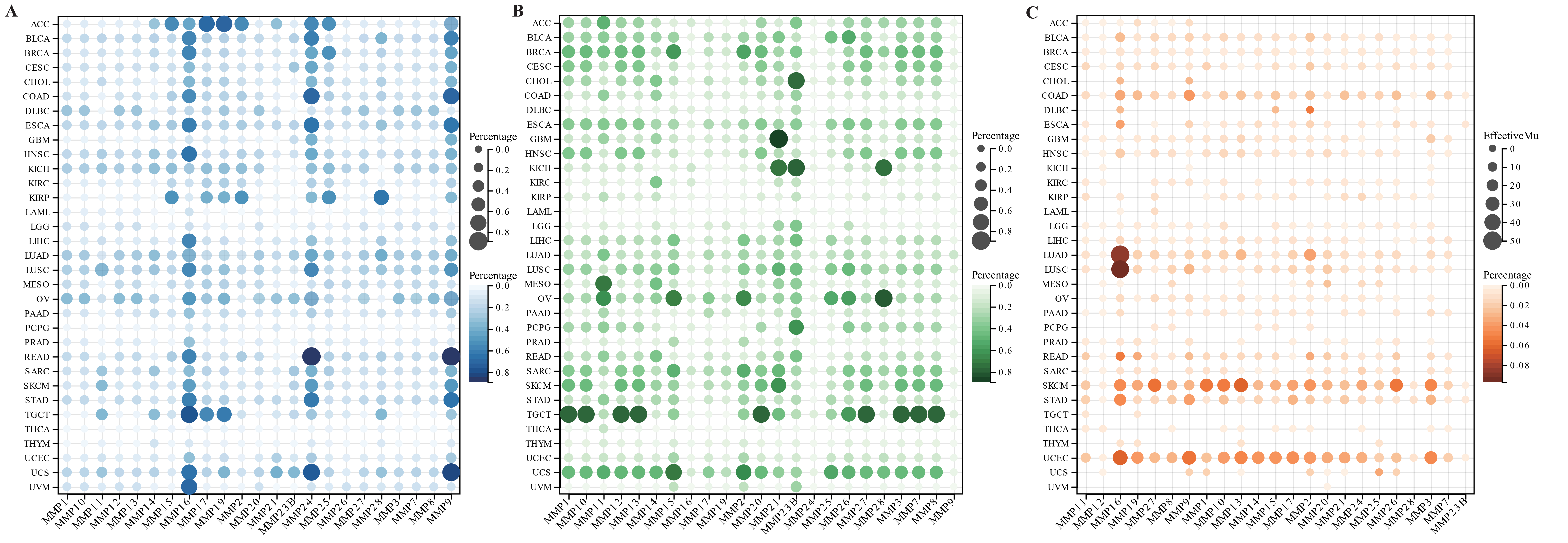

A
B
C
ACC
BLCA
BRCA
CESC
CHOL
COAD
DLBC
ESCA
GBM
HNSC
KICH
KIRC
KIRP
LAML
LGG
LIHC
LUAD
LUSC
MESO
OV
PAAD
PCPG
PRAD
READ
SARC
SKCM
STAD
TGCT
THCA
THYM
UCEC
UCS
UVM
MMP1
MMP10
MMP11
MMP12
MMP13
MMP14
MMP15
MMP16
MMP17
MMP19
MMP2
MMP20
MMP21
MMP23B
MMP24
MMP25
MMP26
MMP27
MMP28
MMP3
MMP7
MMP8
MMP9
Percentage
0.0
0.2
0.4
0.6
0.8
EffectiveMu
0
10
20
30
40
50
0.00
0.02
0.04
0.06
0.08

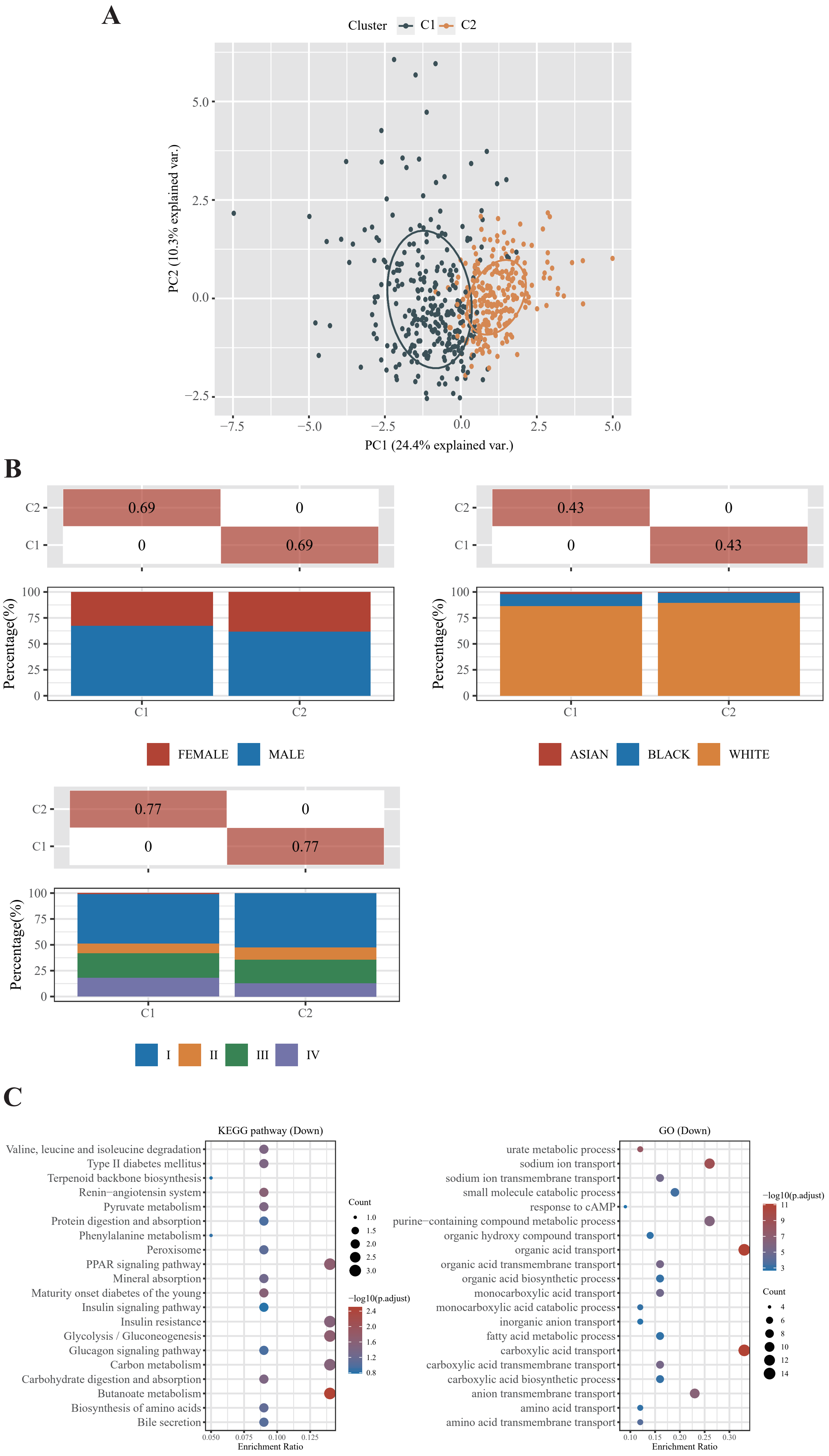
A
Cluster
C1
C2
PC2 (10.3% explained var.)
PC1 (24.4% explained var.)
5.0
2.5
0.0
−2.5
−7.5
−5.0
−2.5
0.0
2.5
5.0
B
C2
C1
0.69
0
0
0.69
Percentage(%)
100
75
50
25
0
FEMALE
MALE
0.43
0
0
0.43
ASIAN
BLACK
WHITE
0.77
0
0
0.77
I
II
III
IV
C
KEGG pathway (Down)
Valine, leucine and isoleucine degradation
Type II diabetes mellitus
Terpenoid backbone biosynthesis
Renin−angiotensin system
Pyruvate metabolism
Protein digestion and absorption
Phenylalanine metabolism
Peroxisome
PPAR signaling pathway
Mineral absorption
Maturity onset diabetes of the young
Insulin signaling pathway
Insulin resistance
Glycolysis / Gluconeogenesis
Glucagon signaling pathway
Carbon metabolism
Carbohydrate digestion and absorption
Butanoate metabolism
Biosynthesis of amino acids
Bile secretion
Enrichment Ratio
Count
−log10(p.adjust)
GO (Down)
urate metabolic process
sodium ion transport
sodium ion transmembrane transport
small molecule catabolic process
response to cAMP
purine−containing compound metabolic process
organic hydroxy compound transport
organic acid transport
organic acid transmembrane transport
organic acid biosynthetic process
monocarboxylic acid transport
monocarboxylic acid catabolic process
inorganic anion transport
fatty acid metabolic process
carboxylic acid transport
carboxylic acid transmembrane transport
carboxylic acid biosynthetic process
anion transmembrane transport
amino acid transport
amino acid transmembrane transport
Enrichment Ratio
−log10(p.adjust)
Count

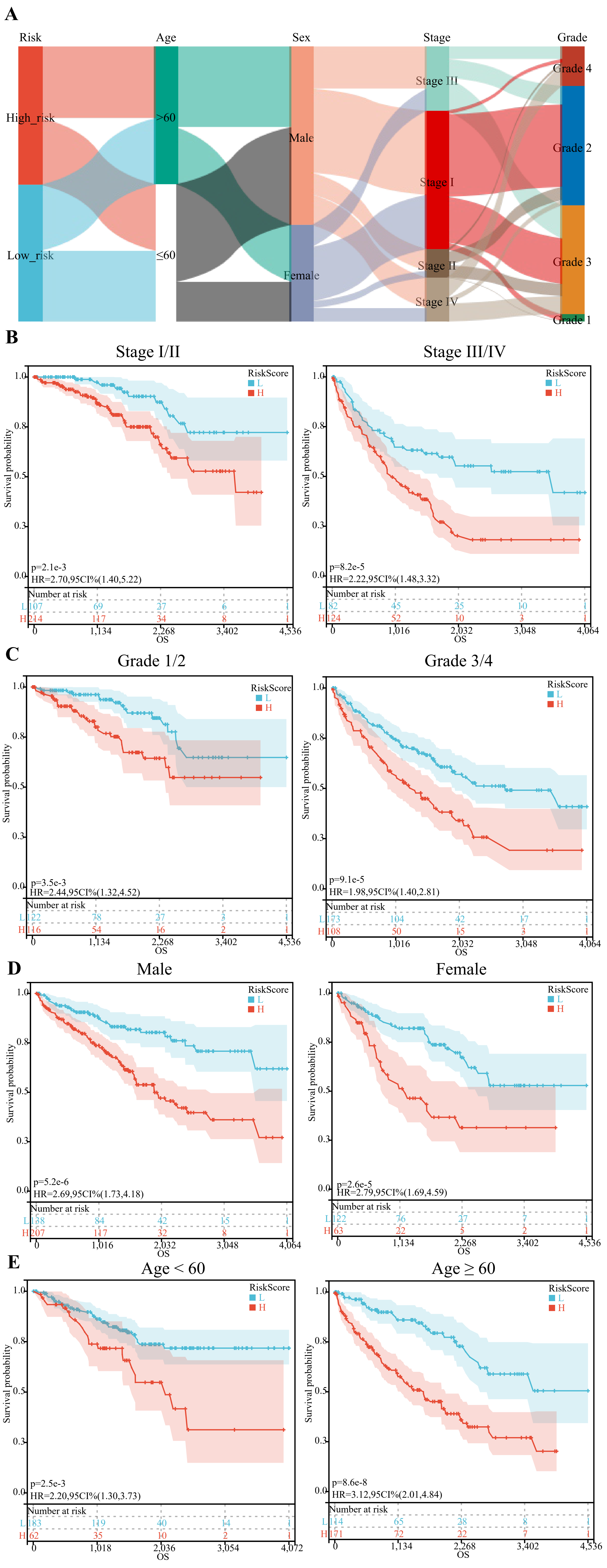
A
Risk
Age
Sex
Stage
Grade
High_risk
Low_risk
>60
≤60
Male
Female
Stage III
Stage I
Stage II
Stage IV
Grade 4
Grade 2
Grade 3
Grade 1
B
Stage I/II
RiskScore
L
H
Survival probability
p=2.1e-3
HR=2.70,95CI%(1.40,5.22)
Number at risk
L 107 69 27 6 1
H 214 117 34 8 1
0 1,134 2,268 3,402 4,536
OS
Stage III/IV
p=8.2e-5
HR=2.22,95CI%(1.48,3.32)
Number at risk
L 82 45 25 10 1
H 124 52 10 3 1
0 1,016 2,032 3,048 4,064
C
Grade 1/2
p=3.5e-3
HR=2.44,95CI%(1.32,4.52)
Number at risk
L 122 78 27 3 1
H 116 54 16 2 1
0 1,134 2,268 3,402 4,536
Grade 3/4
p=9.1e-5
HR=1.98,95CI%(1.40,2.81)
Number at risk
L 173 104 42 17 1
H 108 50 15 3 1
0 1,016 2,032 3,048 4,064
D
Male
p=5.2e-6
HR=2.69,95CI%(1.73,4.18)
Number at risk
L 158 84 42 15 1
H 207 117 32 8 1
0 1,016 2,032 3,048 4,064
Female
p=2.6e-5
HR=2.79,95CI%(1.69,4.59)
Number at risk
L 122 76 27 7 1
H 63 22 5 2 1
0 1,134 2,268 3,402 4,536
E
Age < 60
p=2.5e-3
HR=2.20,95CI%(1.30,3.73)
Number at risk
L 183 119 40 14 1
H 62 35 10 2 1
0 1,018 2,036 3,054 4,072
Age ≥ 60
p=8.6e-8
HR=3.12,95CI%(2.01,4.84)
Number at risk
L 114 65 28 8 1
H 171 72 22 7 1
0 1,134 2,268 3,402 4,536

A

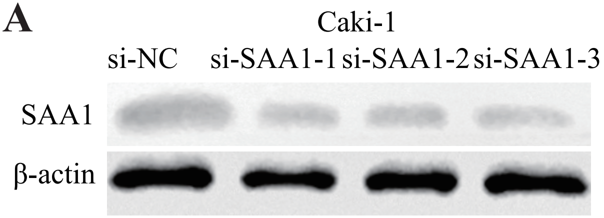

B

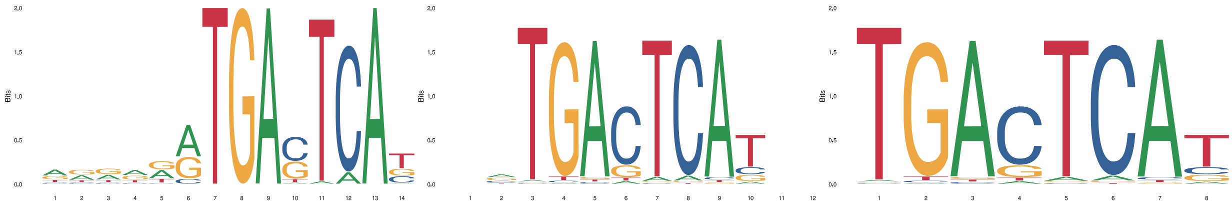

JUN binding site sequence logo

C

Binding sites in MMP7 promoter sequences predicted by JASPAR

TF name	Relative score	Start	End	Strand	Predicted sequence
JUN	0.912	1928	1941	+	CTCAAATGAGTCAC
JUN	0.910	1932	1943	+	AATGAGTCACCT
JUN	0.906	1934	1941	+	TGAGTCAC

Binding sites in MMP9 promoter sequences predicted by JASPAR

TF name	Relative score	Start	End	Strand	Predicted sequence
JUN	0.903	340	347	+	TGACTCAG
JUN	0.903	1922	1929	+	TGACTCAC

Binding sites in MMP17 promoter sequences predicted by JASPAR

TF name	Relative score	Start	End	Strand	Predicted sequence
JUN	0.912	501	510	+	ATGAGGCCAG

Binding sites in MMP13 promoter sequences predicted by JASPAR

TF name	Relative score	Start	End	Strand	Predicted sequence
JUN	0.970	1944	1957	+	AAGTGATGACTCAC
JUN	0.956	1948	1959	+	GATGACTCACCA
JUN	0.955	1950	1957	+	TGACTCAC

Binding sites in MMP19 promoter sequences predicted by JASPAR

TF name	Relative score	Start	End	Strand	Predicted sequence
JUN	0.926	204	217	+	CAAGTGTGAGTCAC
JUN	0.908	208	219	+	TGTGAGTCACCA
JUN	0.906	210	217	+	TGAGTCAC
JUN	0.906	1926	1933	+	TGAGTCAC
JUN	0.902	1924	1935	+	AGAGGAA
